# Genotype network intersections promote evolutionary innovation

**DOI:** 10.1371/journal.pbio.3000300

**Published:** 2019-05-28

**Authors:** Devin P. Bendixsen, James Collet, Bjørn Østman, Eric J. Hayden

**Affiliations:** 1 Biomolecular Sciences Graduate Programs, Boise State University, Boise, Idaho, United States of America; 2 Department of Biological Science, Boise State University, Boise, Idaho, United States of America; 3 Keck Graduate Institute, Claremont, California, United States of America; University of Bath, UNITED KINGDOM

## Abstract

Evolutionary innovations are qualitatively novel traits that emerge through evolution and increase biodiversity. The genetic mechanisms of innovation remain poorly understood. A systems view of innovation requires the analysis of genotype networks—the vast networks of genetic variants that produce the same phenotype. Innovations can occur at the intersection of two different genotype networks. However, the experimental characterization of genotype networks has been hindered by the vast number of genetic variants that need to be functionally analyzed. Here, we use high-throughput sequencing to study the fitness landscape at the intersection of the genotype networks of two catalytic RNA molecules (ribozymes). We determined the ability of numerous neighboring RNA sequences to catalyze two different chemical reactions, and we use these data as a proxy for a genotype to fitness map where two functions come in close proximity. We find extensive functional overlap, and numerous genotypes can catalyze both functions. We demonstrate through evolutionary simulations that these numerous points of intersection facilitate the discovery of a new function. However, the rate of adaptation of the new function depends upon the local ruggedness around the starting location in the genotype network. As a consequence, one direction of adaptation is more rapid than the other. We find that periods of neutral evolution increase rates of adaptation to the new function by allowing populations to spread out in their genotype network. Our study reveals the properties of a fitness landscape where genotype networks intersect and the consequences for evolutionary innovations. Our results suggest that historic innovations in natural systems may have been facilitated by overlapping genotype networks.

## Abstract 2

The determination of the empirical fitness landscape at the genotypic intersection between two different catalytic RNA (ribozyme) functions reveals details about how novel traits can emerge through evolutionary innovation.

## Introduction

The mechanisms by which evolution produces new functions have intrigued biologists since the earliest formulations of evolutionary theory [1,2]. From one perspective, random genetic changes and natural selection for an existing function could prevent novelty if this process were to keep populations near genotypes at the peaks of fitness landscapes and preserve existing forms at the expense of novel mutants [3–6]. Models to explain the origins of new functions often invoke gene duplication events, which create the redundancy needed to allow either copy to eventually evolve toward a new function [7–10]. However, the fitness landscape between old and new functions has been difficult to study largely because of the vast number of possible genetic variants for any given gene. As a result, models of innovation differ in the relative importance of neutral drift, environmental changes, the timing and type of selection pressure, and the high-dimensional nature of sequence space [11]. Our understanding of innovations will benefit from direct observations of the evolution of new structures and functions [12–18].

**Fig 1:**
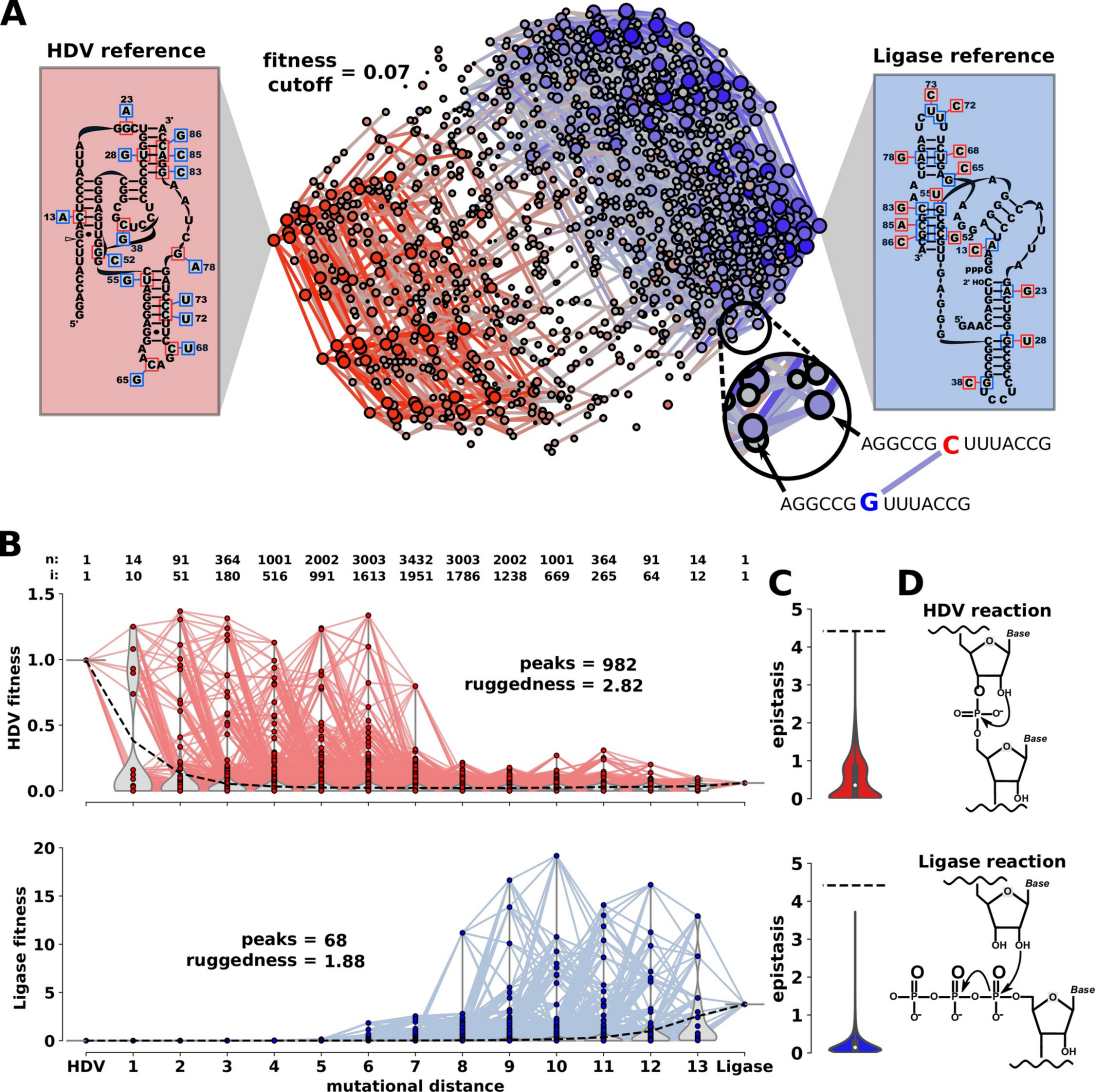
The experimental fitness landscape at the intersection of two genotype networks. (A) Overlay of the HDV and Ligase genotype networks. Nodes represent individual genotypes that are connected by an edge if they are different by a single nucleotide change. Nodes are colored based on their dominant activity (red = HDV; blue = Ligase). For each genotype, “ribozyme fitness” is defined as the relative ribozyme activity determined by high-throughput sequencing and is indicated by the size of the node and the color saturation. Genotypes with fitness below 0.07 are excluded for visualization purposes. Boxes on the left (HDV reference) and right (Ligase reference) show the secondary structure for the reference genotypes and all the mutational changes that were analyzed. The mutations in blue boxes convert the HDV reference to the Ligase reference. The mutations in red boxes convert the Ligase reference to the HDV reference. (B) Distance-based layout of the two fitness landscapes. Each sequence is positioned on the x-axis according to its mutational distance from the HDV reference genotype. HDV fitness (red) and Ligase fitness (blue) are indicated by the y-axis value. The number of genotypes (*n*) increases in the middle of the plot, and the total number of genotypes at each position is indicated about the graph. The number of dual-function intersection sequences (i) at each mutational distance is also indicated. Inset text “peaks” and “ruggedness” describe quantitative characteristics of the landscapes. Data and Python scripts used to construct fitness landscapes can be found on GitLab. (C) Distributions of the magnitude of epistatic values found in each landscape. Data and Python scripts used to calculate and graph epistasis can be found on GitLab. (D) Representation of the chemical reactions catalyzed by each ribozyme. HDV, Hepatitis Delta Virus.

Macromolecular phenotypes such as the activity of enzymes can tolerate changes to their primary sequence (mutations) without necessarily changing structure or function. Many genotypes (sequences) have the same phenotype (enzymatic activity) [19,20]. Natural populations of both organisms and macromolecules that appear the same phenotypically still harbor many genetic differences. Genotype networks are the collection of all genotypes with the same phenotype that are interconnected by mutational steps [21]. The expansiveness of genotype networks provides robustness because mutations are likely to preserve the existing phenotype. However, it has also been argued that genotype networks can facilitate evolutionary innovation because different regions of the vast genotype networks provide mutational access to new structures and functions. Populations occupy finite regions of these vast networks, and it has been suggested that innovations can occur when populations encounter regions of genotype space where two different genotype networks are in close proximity [22] (Fig 1A). In recent years, experimental advancements have enabled extensive mapping of genotype to phenotype. However, with few exceptions, these mappings have been used to understand the fitness landscape of a single function. In order to evaluate the innovation potential of genotype networks, it is necessary to characterize the number of mutations that separate two different genotype networks and the fitness consequences of the mutational changes needed to move from one network to the other.

Here, we report an experimentally constructed “ribozyme fitness” landscape at the intersection of two genotype networks. For our study system, we have chosen two distinct RNA phenotypes. The RNA molecules are ribozymes, structured noncoding RNA molecules that catalyze chemical reactions. One ribozyme phenotype is the naturally occurring self-cleaving Hepatitis Delta Virus (HDV) ribozyme. The second phenotype is the class III Ligase ribozyme that was discovered through artificial selection in a lab [23,24]. The two ribozymes fold into very different structures (Fig 1A) and catalyze different chemical reactions (Fig 1D). Despite the differences between the two ribozymes, it was previously shown that the two genotype networks come in close proximity, and very few mutations could convert one ribozyme into the other [24]. This provides an experimentally tractable example of a molecular innovation. To characterize the effects of mutations required to move between the two genotype networks, we developed two high-throughput-sequencing–based assays to quantify both ribozyme phenotypes. Although the two prototype ribozyme sequences are separated by 67 mutations, we identified two reference genotypes with approximately “wild-type” levels of activity that contained 14 mutational differences between them. We synthesized DNA templates needed to transcribe the RNA molecules that contain all the combinations of these mutational differences. We analyzed the 2^14^ = 16,384 neighboring RNA sequence variants using both ribozyme assays (S1 Fig). For each sequence, we determined the ribozyme fitness for both activities, defined as the performance of the sequence in our assays relative to a reference sequence. For the HDV phenotype, our ribozyme fitness is defined by the fraction of the sequence that self-cleaves during transcription. For the Ligase phenotype, ribozyme fitness is defined as the change in abundance of each sequence from a single round of selection for Ligase activity (see Materials and Methods).

We, like others, use performance in an in vitro assay as a proxy for fitness [25–28], and we do not provide experimental confirmation that the ribozymes studied here alter the fitness of any organism. We note that there are several examples of a simple correlation between enzyme activity and organismal fitness [29,30], and our simulation based analysis that follows assumes such. However, the relationship between the properties of gene products and organismal fitness is typically complex, often environmentally dependent, and the subject of numerous lines of investigation [31,32]. Because of the vastness of sequence space, the ability to predict evolutionary outcomes in the lab and in natural environments will require advancements in high-throughput in vivo and in vitro assays, as well as in computational approaches to merge data across scales. In this spirit, we used our in vitro determined ribozyme fitness values to analyze the billions of mutational trajectories between the two genotype networks and use computational simulations to explore how these proximal genotype networks might impact evolutionary innovations.

## Results

### Empirical ribozyme fitness landscape at the intersection of two genotype networks

We obtained ribozyme fitness measurements for all 16,384 RNA sequences for both RNA phenotypes. For visualization of the resulting genotype networks, we plot the data as a network graph, in which each node is a unique sequence, nodes are connected if they differ by a single mutation, and the fitness is represented by the size and color saturation of the node (Fig 1). Each node is colored based on the dominant activity, with HDV in red and Ligase in blue. Fitness values were normalized such that fitness = 1 for the reference ribozyme, previously referred to as the “prototype” [24]. This representation of the data allows a visual appraisal of the proximity of the two genotype networks. In general, both networks are characterized by a decrease in fitness with distance from the reference. The region where the two networks are in closest proximity contains sequences with low activity for both functions. Still, we find that numerous genotypes in the two networks are proximal, creating numerous mutational trajectories between the two functions. Characterizing the mutational distance between the two networks requires numerous distance measurements.

### Proximity and functional overlap of the two genotype networks

**Fig 2:**
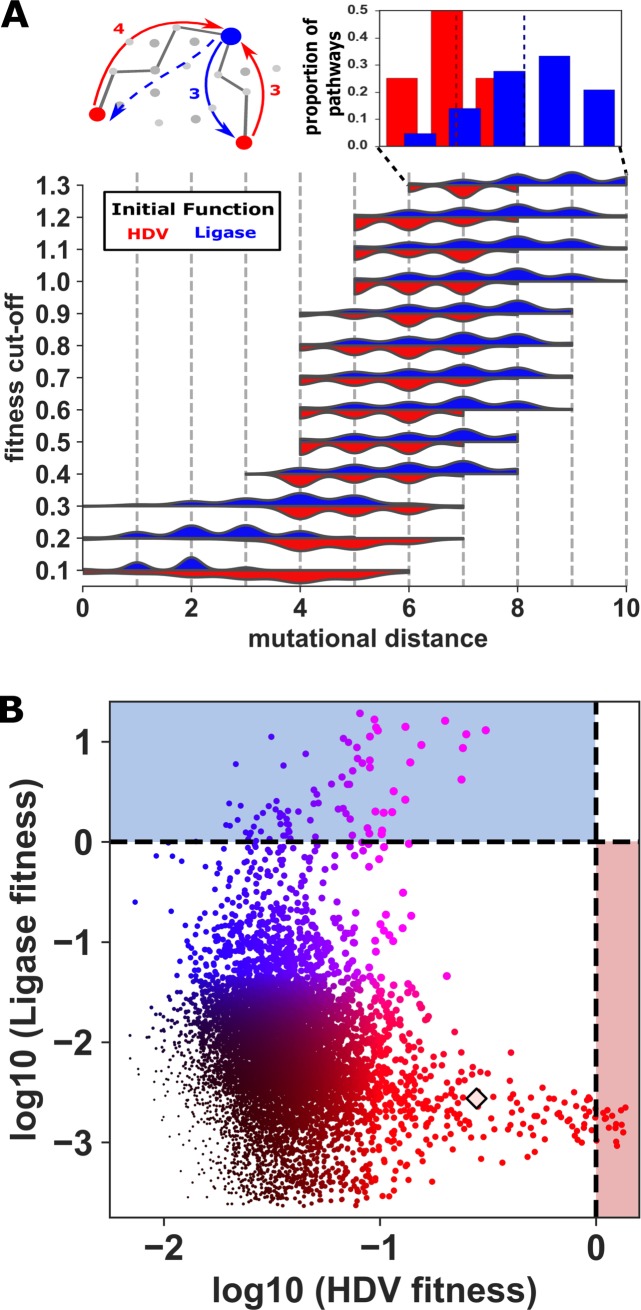
Proximity and overlap of the two genotype networks. (A) Distributions of shortest mutational distance (x-axis) between genotypes on different networks as a function of fitness cutoff (y-axis; blue = Ligase to HDV distances; red = HDV to Ligase distances). For each genotype with a fitness above the cutoff value for one function, the distance to the nearest genotype with the other function was determined. The distribution of these distances determined for all genotypes are plotted as violin plots. The diagram (above, left) illustrates the measurement of distance between the two functions. Inset (above, right) shows the distribution at fitness cutoff = 1.3 as histograms, and dashed lines indicate the sample means. Data and Python scripts used to plot distributions can be found on Gitlab. (B) Intersection sequences with detectable activity for both functions. For each genotype, the HDV fitness is plotted on the x-axis, and Ligase fitness is plotted on the y-axis. Color indicates the ratio of Ligation fitness (blue) to HDV fitness (red). The size of the node is scaled to the higher of the two fitness values. Fitness values are log10 transformed. Dashed lines indicate wild-type level activity with fitness = 1 (log10 fitness = 0). Data and Python scripts used to plot intersection sequences can be found on Gitlab. HDV, Hepatitis Delta Virus.

To quantify the average distance between the two genotype networks, we measured the mutational distance between every genotype on one network and the nearest genotype on the other network with equivalent or greater fitness (Fig 2A). We found that this distance depends upon whether or not a lower bound is set for genotypes to be considered a member of the genotype network. We found that the average distance between the networks decreased as the fitness cutoff is lowered (Fig 2A). For example, if “wild-type” activity is required (fitness > 1), the two networks are separated by approximately 7 mutations on average (S2 Fig). However, if molecules with 10% of wild-type activity or better are considered part of the network, then most genotypes are only 1–2 mutations from the other network. We found that the number of connections between the two networks was also dependent upon the fitness cutoff. Specifically, decreasing the fitness cutoff increased the connectivity between the networks (S3 Fig).

Interestingly, when no minimal fitness cutoff is imposed, we find that numerous genotypes appear to catalyze both reactions, each representing a point of intersection between the two genotype networks (Fig 2B). We expected to find some dual-function intersection sequences because we intentionally designed our library around the sequence space of a previously discovered dual-function sequence. With a maximum of 14 mutations between all the sequences, it was not surprising that most sequences maintained one of the functions. However, the number of potential dual-function sequences in our data was surprising. Admittedly, the precise number of these dual-function intersection sequences is difficult to determine because many sequences show very low activity for one of the functions that is near the limits of our detection at the sequencing depth achieved. We therefore set a cutoff that each specific sequence must be detected as active at least 3 times in our data, once in each replicate. Based on the high quality of our sequence data (S4B Fig), we predict that it is very unlikely that sequencing errors from mutational neighbors could produce a false positive with this cutoff because it would require a precise sequence error 3 separate times. Based on this cutoff, we find that over half the genotypes (9,032) can perform both functions (Fig 2B). Specifically, we detected HDV activity for 9,032 of the genotypes and Ligase activity for 16,384 genotypes.

We took several steps to confirm that low-fitness genotypes were in fact active ribozymes. First, we carried out in vitro assays of self-cleavage and ligation activity for several genotypes. We determined self-cleavage activity by gel electrophoresis and Ligase activity by quantitative PCR (see Materials and Methods). Both assays supported our sequence-based fitness measurements (S5 Fig). However, these in vitro methods are less accurate for low-fitness sequences, which required further investigation. Therefore, we also compared the read counts of the lowest-fitness sequences to counts of spurious sequences that were not intentionally synthesized in our library. These spurious genotypes had mutations outside the 14 variable nucleotide positions. The least frequent HDV genotypes in our data that showed self-cleavage activity were observed as cleaved more than once in all 3 replicates and uncleaved more than 108 times (S6 Fig). In contrast, spurious reads were typically only observed once, either as cleaved or uncleaved (S7 Fig). We note that genotypes that were not detected as cleaved in any individual replicate were not considered active. The lowest-fitness Ligase genotype was observed as ligated more than 4 separate times in a given replicate and more than 32 times across all 3 replicates, again in contrast to the rarity of spurious reads. Further, for all sequences, we also estimated enzymatic ligation rates from our fitness measurements and compared these rates to reported nonenzymatic ligation rates. To accomplish this, we identified a genotype that was in the original intersection sequence study [24] and in our current data and assumed that this ribozyme had the same enzymatic rate in both studies. We then converted our fitness values to rates using a linear transformation. This estimated ligation rate indicated that all of the Ligase measurements in our study were above the template-directed, nonenzymatic oligonucleotide ligation rate (S8C Fig). Finally, a positive correlation between frequency and fitness would be expected if our selection for ligation activity allowed random sequences to pass through without actually catalyzing a ligation (often termed a “leaky” selection). However, we found no correlation (S8 Fig).

Most of the identified dual-function intersection sequences have very low fitness for both functions, and not surprisingly, no single sequence had higher than wild-type fitness for both functions (log_10_(fitness) > 0). However, several sequences did show detectable levels of activity for one function and higher than wild-type fitness for the other function. Under many evolutionary scenarios, these genotypes could be the most likely to facilitate a molecular innovation because they could persist in a population if selection was acting on only one function yet would already provide the new function as a suboptimal promiscuous function [33,34].

### Computational simulations of evolutionary innovation on the empirical fitness landscape

**Fig 3:**
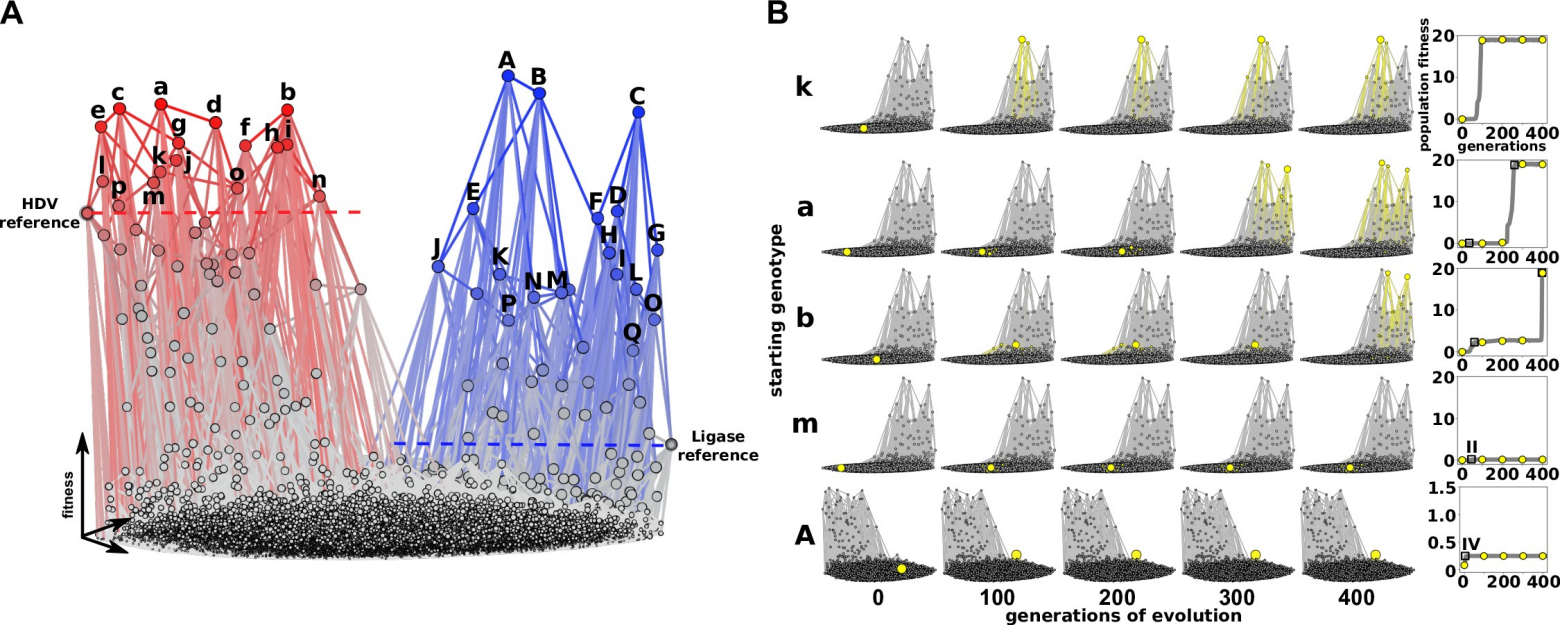
Periods of evolutionary stasis revealed by computational simulation of evolutionary innovation. (A) A landscape visualization of the two genotype networks. The height of each node (z-axis) indicates the relative fitness for the HDV phenotype (red) and the Ligase phenotype (blue). Nodes represent genotypes and are connected by an edge if they are different at one nucleotide position. Fitness is indicated by the height (z-axis), the size of the node, and the color saturation. Fitness values are normalized so that both graphs are similar heights. Genotypes used to start evolutionary simulations are labeled with lower case for the genotypes with the highest HDV fitness (a–p) and capital letters for genotypes with the highest Ligase fitness (A–Q). (B) Frames from simulations of evolving populations. Several examples are shown to illustrate different rates of increase of “population fitness” over simulation time (“generations”). Each row shows the progress of a single simulation. The starting genotype is indicated to the left. Each plot shows the genotypes present in the population with the number of generations of evolution labeled at the bottom. Genotypes present in the population are indicated by yellow nodes and edges. The corresponding mean fitness of each population over time is shown in the plots to the right. During simulations, the population size (*N* = 1,000) and mutation rate (*μ* = 0.01) were constant. HDV, Hepatitis Delta Virus.

Next, we set out to evaluate the implications of these genotype networks for the evolution of molecular innovations. The networks are high-dimensional, which limits any intuitive interpretation. We therefore turned to computational simulations of populations of RNA molecules evolving on the networks. We modeled evolution using a Wright–Fisher model [35] with a fixed population size, a fixed mutation rate, and a probability of survival determined by the experimental relative ribozyme fitness values for each genotype (see Materials and Methods). For these simulations, it is useful to visualize the genotype networks as a landscape where the height of the landscape is determined by the fitness (Fig 3A and S1 Movie). In our simulations, evolving populations will tend to move uphill toward peaks, defined as sequences where all 1-mutation neighbors have lower fitness. The crossing of fitness valleys to get from low-fitness peaks to higher-fitness peaks is allowed in our simulations but requires a stochastic series of less likely events. We applied this evolutionary simulation to three scenarios of evolutionary innovation: 1) immediate selection for the new function following gene duplication, 2) neutral evolution prior to selection for the new function, and 3) simultaneous selection for both functions. This last scenario represented evolutionary innovation prior to gene duplication.

### Immediate selection for the new function following gene duplication

The first scenario modeled evolution following a gene duplication event, in which a new copy of a gene was under selection for a new function and the other copy simply maintained the original function. We therefore only followed the evolution of the new function for this scenario. We applied immediate selection pressure for the new function, with no consequence for the changes in the initial function. This scenario was simulated in both directions, with either Ligase or HDV functions representing the new function.

**Fig 4:**
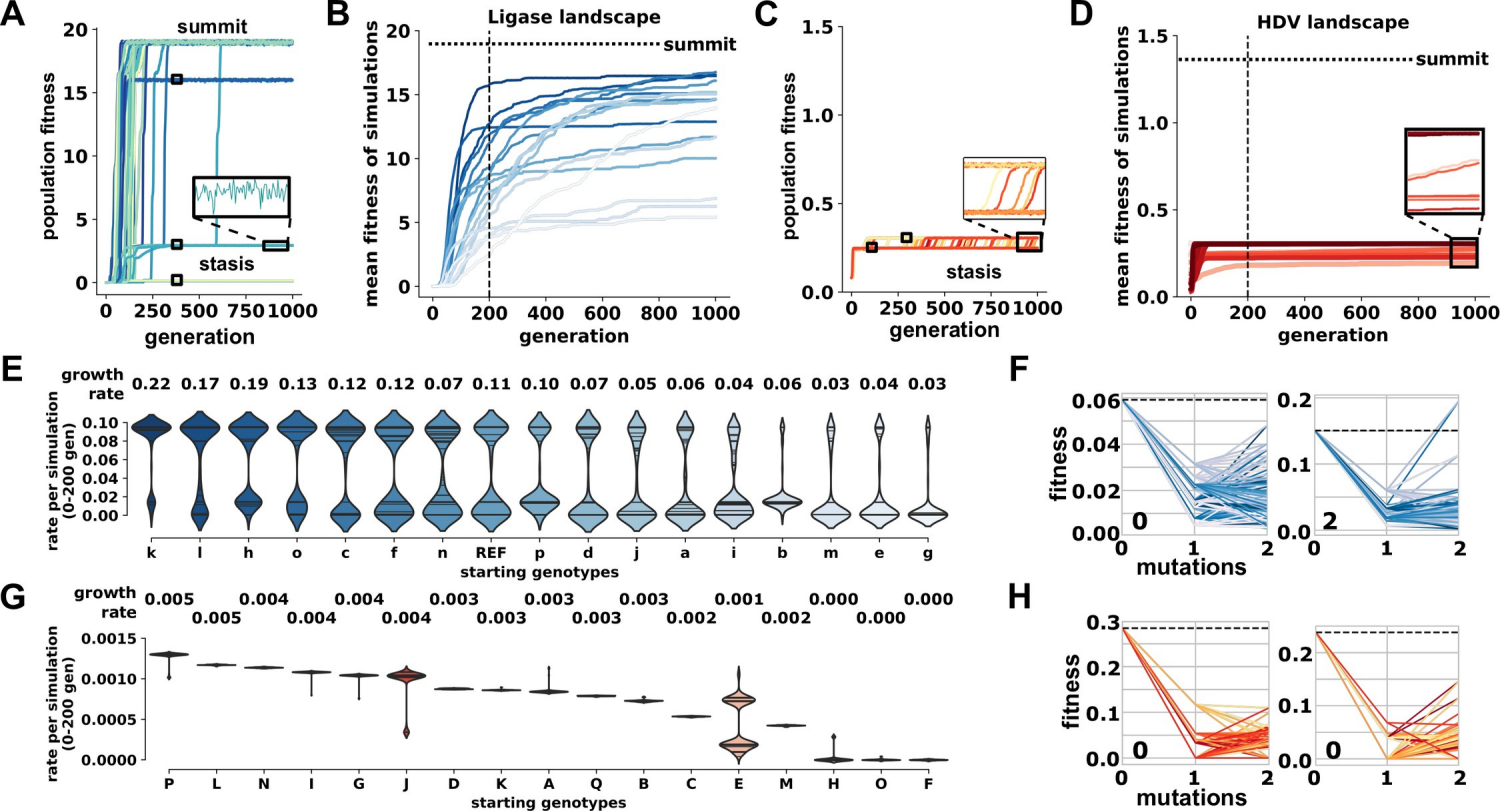
Starting genotypes result in different rates of evolutionary adaptation. (A) Rates of Ligase adaptation from a single HDV genotype. Each trace shows the average population fitness as a function of generation time for a separate simulation of 1,000 individuals each. The traces from 100 separate simulations are shown. Inset shows minor fluctuations during periods of stasis. Data and Python scripts for evolutionary simulations can be found on GitLab. (B) Average rates of evolutionary adaptation of Ligase activity starting from 17 genotypes. Each trace represents a different starting genotype (a–p and HDV reference) and shows the mean fitness of 100 simulations as a function of time (“generation”). The y-axis is scaled to the maximum fitness on this landscape (“summit,” horizontal dashed line). The vertical dashed line marks generation 200. Data and Python scripts for evolutionary simulations can be found on GitLab. (C) Rates of HDV adaptation from a single Ligase genotype. Data and Python scripts for evolutionary simulations can be found on GitLab. (D) Average rates of evolutionary adaptation of HDV activity starting from 17 genotypes. Each trace represents a different starting genotype (A–Q) and shows the mean fitness of 100 simulations as a function of time (“generation”). The y-axis is scaled to the maximum fitness on this landscape (“summit,” horizontal dashed line). Data and Python scripts for evolutionary simulations can be found on GitLab. (E) Distributions of initial rates of adaptation during simulations on the Ligase landscape. Initial rate is determined as the population fitness divided by the generations at 200 generations. Each violin plot represents the distribution of 100 simulations starting from the same genotype, which is indicated on the x-axis. Maximum growth rate, determined from a cubic spline regression, is also reported. Growth rate calculations and plots are reported in S10 Fig. Data and Python scripts for evolutionary simulations can be found on GitLab. (F) Sign epistasis in the local fitness landscape of genotypes that cause periods of stasis in the Ligase landscape. The fitness of the stasis genotype is plotted at mutations = 0, and this starting fitness is marked with a dashed line. The fitness of neighboring genotypes that differ by 1 or 2 mutations are shown. Distributions of initial rates of adaptation during simulations on the HDV landscape. Data and Python scripts for plotting local fitness landscapes can be found on GitLab. (G) Distributions of initial rates of adaptation during simulations on the HDV landscape. Growth rate calculations and plots are reported in S16 Fig. Data and Python scripts for evolutionary simulations can be found on GitLab. (H) Sign epistasis in the local fitness landscape of genotypes that cause periods of stasis in the HDV landscape. Data and Python scripts for plotting local fitness landscapes can be found on GitLab. HDV, Hepatitis Delta Virus; REF, reference.

We started multiple simulations, each from different genotypes on the HDV network, and challenged the populations to evolve on the Ligase fitness landscape. The starting genotypes selected all had above wild-type HDV fitness and therefore would be likely to persist in a population under selection for the HDV function. We recorded these simulations as movies to observe the process of evolution toward the new Ligase function (Fig 3B and S2–S5 Movie). We noticed that many of the individual simulations had periods during which the mean fitness of the population plateaus at a specific, often low value for many generations (Fig 3B). To evaluate the average contribution of these periods of stasis, we repeated the simulation 100 times and plotted the average fitness of the evolving population over time (Figs 4A and S9). We carried out 100 replicates each for all of the different starting genotypes (Fig 4B and S2 Table). We found that different genotypes on the HDV network resulted in consistently different average rates of adaptation to the new Ligase function (Fig 4E). The maximum growth rate derived from the regression analysis for each starting genotype found similar results (S10 Fig). It is important to note that the rate of adaptation was not dependent or correlated with the mutational distance between the starting and summit genotypes (S11 Fig).

Additionally, we found that there exist specific genotypes on the Ligase fitness landscape that caused these periods of stasis and slower average rates of adaptation (Figs 4F and S12 and S13). These genotypes are local peaks that are characterized by very few pathways to higher fitness. Importantly, the genotypes that caused the longest periods of stasis and slowest rates of adaptation are characterized by extensive reciprocal sign epistasis, meaning that achieving higher fitness requires two or more mutational steps, but every initial step is deleterious. Specific starting genotypes on the HDV network frequently stalled at the same intermediate fitness level, indicating that they were likely to encounter a specific stasis-causing fitness peak. It is important to note that the dynamics of our simulations are not significantly altered by the accuracy of low-fitness sequences. This is because the rate of adaptation is dominated by the local fitness peaks that are surrounded by genotypes with very low fitness, but the precision of our fitness measurements for these low-fitness genotypes does not alter our evolutionary dynamics. As evidence, we observed nearly identical evolutionary outcomes when simulations were repeated after 7,015 genotypes with fitness < 0.005 were converted to fitness = 0 (S14 Fig). Overall, the consistent rates of adaptation from multiple simulations are encouraging for efforts aimed at forecasting evolutionary outcomes, especially in cases in which the underlying fitness landscape can be measured or accurately estimated [26,36].

We next repeated the evolutionary simulations from the opposite perspective, starting with genotypes from the Ligase side of the landscape with selection for improved HDV function. This scenario models Ligase as the original function and HDV self-cleavage as the new function that is under selection following gene duplication. Surprisingly, we found that all of these simulations got stuck at very low fitness for the full 1,000 generations (Figs 4C and 4D and S15), resulting in significantly slower rates of adaptation (Fig 4G). The maximum growth rate derived from the regression analysis for each starting genotype found similar results (S16 Fig). We note that the simulations were done under identical population size and mutation rate, and we therefore attribute the different evolutionary dynamics to properties of the fitness landscapes. The property identified that was likely to dictate evolutionary dynamics was the ruggedness of the landscape. We find that the HDV landscape is much more rugged than the Ligase landscape, with more peaks and more extensive sign epistasis. The HDV landscape has 982 peaks, while the Ligase landscape has only 68, which is caused by more frequent instances of sign epistasis in the HDV landscape. The severity of sign epistasis is also higher on the HDV landscape, which can be seen in the extreme values in Fig 1C.

### Neutral evolution model of evolutionary adaptation

The fact that some genotypes promoted very rapid adaptation supports the idea that neutral evolution that enables a population to explore a genotype network can facilitate evolutionary innovations [22,37]. We next modeled a period of neutral evolution prior to selection for the new function. For these simulations, we allowed increasing amounts of neutral evolution from 0 to 1,000 generations at 100 generation increments. We simulated neutral evolution on both the Ligase and HDV landscape starting from the summit genotype (Fig 4, genotypes a and A) of one landscape prior to evolving under selection for the other function.

**Fig 5:**
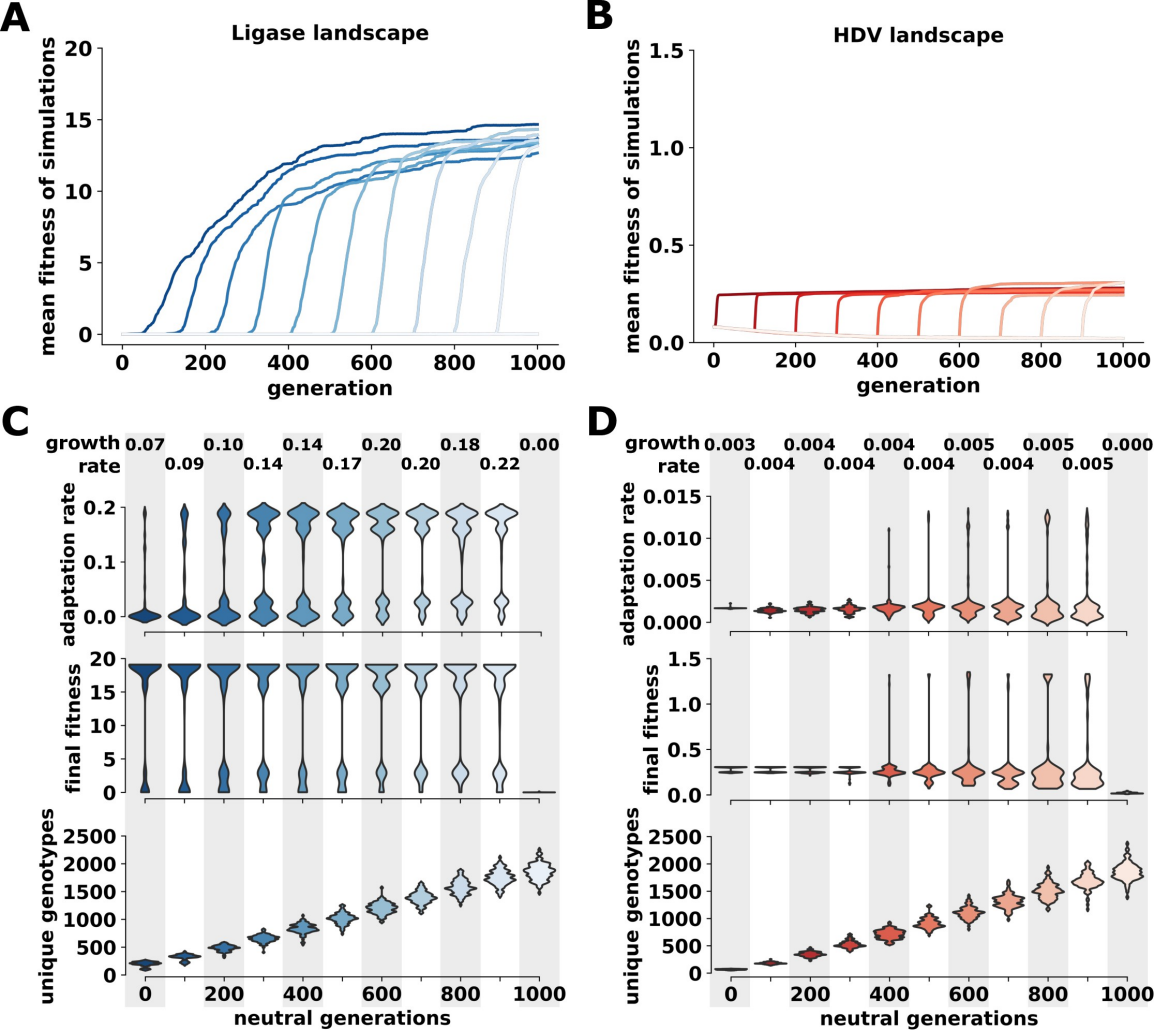
The effects of neutral evolution on evolutionary adaptation. (A) Average rates of evolutionary adaptation of Ligase activity starting from the summit genotype of the HDV landscape. Each trace represents a different number of generations of neutral evolution and shows the mean fitness of 100 simulations as a function of time (“generation”). The y-axis is scaled to the maximum fitness on this landscape (“summit”). The vertical dashed line marks generation 200. Data and Python scripts for evolutionary simulations can be found on GitLab. (B) Average rates of evolutionary adaptation of HDV activity starting from the summit genotype of the Ligase landscape. Data and Python scripts for evolutionary simulations can be found on GitLab. (C) Distributions of rates of adaptation, final population fitness, and the number of unique genotypes explored following generations of neutral evolution during simulations on the Ligase landscape. Each violin plot represents the distribution of 100 simulations following the same length of neutral evolution, which is indicated on the x-axis. Adaptation rate is determined as the rate of population increase for the first 100 generations following the neutral evolution. Final fitness is the mean population fitness at the end of 1,000 generations of evolution. Maximum growth rate, derived from a cubic spline regression, is also reported. Growth rate calculations and plots are reported in S19 Fig. Data and Python scripts for evolutionary simulations can be found on GitLab. (D) Distributions of rates of adaptation, final population fitness, and the number of unique genotypes explored following generations of neutral evolution during simulations on the HDV landscape. Growth rate calculations and plots are reported in S20 Fig. Data and Python scripts for evolutionary simulations can be found on GitLab. HDV, Hepatitis Delta Virus.

As expected, the mean fitness of the populations did not improve during periods of neutral evolution, as indicated by the lag at the beginning of each simulation (Fig 5A and 5B and S17 and S18). However, we found that this period of neutral evolution increased the rate of adaptation toward the new function when selection pressure was applied. We measured this increase in adaptation as either the population fitness in the first 100 generations of selection for the new function (termed adaptation rate) (Fig 5C and 5D) or as the maximum growth rate from a nonlinear regression (S19 and S20 Fig). The adaptive advantage increased with longer periods of neutral evolution. We also found a corresponding increase in the final population fitness obtained (Fig 5C and 5D). Interestingly, following as few as 400 generations of neutral evolution, populations on the HDV landscape were able to reach the HDV summit (S18 Fig and S7 and S9 Movie), which we did not observe without neutral evolution (S15 Fig). As generations of neutral evolution increased, the probability of a population reaching the HDV summit also increased. This trend was also observed on the Ligase landscape, where populations often reach the summit without neutral evolution (S17 Fig and S10 Movie). Although neutral evolution on the HDV landscape allowed some populations to reach the summit, it also trapped some populations at suboptimal peaks with lower fitness than was reached by simulations without neutral evolution. We also found that the neutral evolution increased the number of unique sequences explored by the population (Fig 5C and 5D). We conclude that the period of neutral evolution improved adaptation rates because it allowed the population to fortuitously discover genotypes with easier paths to higher fitness.

### Coselection model of evolutionary adaptation

We also modeled a scenario in which both functions were simultaneously under selection and each function contributed to fitness (S21 and S22 Figs). For these simulations, we assigned each genotype a fitness that was calculated by summing the HDV and Ligase fitness values, each multiplied by an adjustable weighting parameter. Before summing, we normalized the data by dividing each fitness values by the maximum fitness value in that landscape such that the maximum fitness of both functions was fitness = 1. We found that under this scenario, the function that was weighted more heavily would be optimized at the expense of the function with lower weighting. Interestingly, when both functions were given equal weight, the result was stochastic, and either HDV or Ligase could be optimized. We did not observe any instances in which the population remained split with some genotypes being selected for high HDV fitness and others with high Ligase fitness. This suggests that prior to gene duplication, a given population is likely to have genes that are optimized for one function or the other but not both.

## Discussion

The goal of this study was to characterize the ribozyme fitness landscape at the intersection of two genotype networks in order to advance our understanding of the challenges to evolutionary innovations at the molecular level. Until recently, the study of genotype networks was mostly based on computational experiments, such as the thermodynamic prediction of RNA secondary structures [38–40] or simplified models of protein structures [41]. These foundational experiments established that there are numerous genotypes that can produce the same phenotype, and these genotypes are connected by small mutational changes to produce networks that cover vast regions in the space of all possible genotypes. These networks were often referred to as “neutral networks” because they did not assign a fitness value to each genotype and only predicted a structure or “shape.” However, evolution does not act on shape alone. A large body of research has used mathematical models to assign fitness values to genotypes in order to evaluate evolution across genotype networks. Researchers can now use high-throughput assays to assign experimentally determined fitness values to genotypes in order to study the pathways to higher fitness [42–47]. The vast majority of experimental fitness landscapes have focused on a single function. Our results build upon efforts toward looking at the interface of different genotype networks. We can only evaluate how fitness landscapes contribute to evolutionary innovation by focusing on multiple functions [24,48,49].

Using computational simulations, we found several differences in the evolutionary dynamics on each landscape that have implications for evolutionary innovations. For example, our results indicate that the order in which new functions arise can alter evolutionary dynamics because optimizing HDV activity from sequences with high Ligase activity was much more challenging than evolving in the reverse order. In addition, we found that specific properties of the genotype networks dictated the rate of evolutionary adaptation of a new function. The Ligase landscape was less rugged with fewer peaks, which allowed the more rapid evolutionary adaptation of the new function in our simulations. Interestingly, we found that these differences in adaptation rates hold over a range of population sizes and mutation rates (S24 and S25 Figs). This highlights that the severe ruggedness of the HDV landscape cannot be easily overcome by changing model parameters. Periods of neutral evolution may be critical for evolutionary optimization on severely rugged landscapes such as the HDV landscape [50,51]. Several laboratory and theoretical studies have also found that periods of genetic drift or nearly neutral evolution can improve adaptation rates [37,52]. On the other hand, it has been argued that neutral drift is not necessary for crossing valleys in a fitness landscape, and the contribution of neutral drift becomes less significant for the evolution of complex traits involving multiple genes [53]. We observe that the ribozyme innovation studied here can evolve without neutral evolution. Our results support the view that both neutral drift and directional selection play important roles in the evolution of innovation [22], and their relative contribution will depend on specific parameters such as time scales, population sizes, mutation rates, and the underlying fitness landscape [50].

It is important to reiterate that it is difficult to predict how the relative activity of a protein or RNA enzyme will translate to organismal fitness, especially when multiple enzymes interact and are exposed to environmental changes. When experimental and computational advancements enable more extensive mappings of genotype to fitness at the organismal level and across multiple environments, these landscapes may or may not show properties similar to protein and RNA fitness landscapes. We therefore see our evolutionary simulations as a way to characterize multidimensional experimental fitness landscapes that allows for the stochastic events needed to cross fitness valleys. As compared to previous analyses that predict the avoidance of pathways with fitness valleys, our simulations emphasize that fitness peaks, not valleys, dominate adaptation rates. We predict that identifying and characterizing the sequence space around fitness peaks will be necessary to move toward evolutionary predictions at the organismal level [36,54].

Our results provide a glimpse into how intersection sequences promote the evolution of new functions and enable the expansion of biodiversity. The high frequency of dual-function intersection sequences in our data supports the idea that ancient genes that duplicated and enabled radiation events [55] may be characterized by both significant functional overlaps and a robust genotype network. Further investigations into intersection sequences and fitness landscapes will be required to fully evaluate this scenario. For example, our current library design only investigates two nucleotides at each variable position, which represent the parsimonious or “direct” pathways between the two reference genotypes. However, recent experimental evidence from a protein enzyme supports the idea that higher-dimensional “indirect” pathways can bypass epistasis and facilitate adaptation [56]. Further experiments with different library designs will be required to determine how higher-dimensional landscapes contribute to evolutionary innovations [38].

Our results support insights gained from earlier computational studies. For example, one prior computational study of simple RNA secondary structures, termed “shapes,” looked at the most probable new shapes that are 1 mutation away from sequences that form a canonical tRNA structure [39]. The authors found that most single mutations produce very similar shapes. However, they also found that there exist some single mutations that produce shapes with considerable differences. The HDV and Ligase structures in the current study do not share any structural similarity, but our results show that the shapes overlap extensively in sequence space such that there is a high probability of finding one ribozyme in the neighborhood of the other. This similarity between computational and experimental data is somewhat surprising because the ribozyme phenotypes studied here require a precise tertiary structure to achieve catalysis that is not taken into account by secondary structure prediction. Nevertheless, the canonical base pairing interactions that are computationally predicted make up a large component of the structural interactions needed for ribozyme folding, which may account for the similarity between the results. Regardless, our results support the long-standing use of computational prediction of RNA structures as a realistic model of the genotype-to-phenotype relationship, which continues to inspire experiments. This also provides motivation for continued efforts to use experimental structure probing methods to improve the blind prediction of RNA tertiary structure [57].

The decrease in the fitness of both functions at the intersection suggests that intermediate forms are evolutionarily disfavored over the sequences that can do one function well [11]. The evolution of innovation in this sequence space is therefore not only possible but probable because, once a population discovers this region of sequence space, selection is likely to favor a genotype with one function or the other. For example, we found that populations were about equally likely to optimize either function when both functions were simultaneously under selection and contributed equally to fitness. The importance of environmental changes should not be overlooked. A sudden environmental shift could quickly favor one function over the other, and a fluctuating environment could alter selection pressures and help maintain both functions [58,59].

It remains unknown whether these characteristics are common or peculiar to the specific phenotypes investigated here. Further research advancements will be required to study larger expanses of genotype space needed to cover more mutational positions and the resulting higher dimensionality. It will also be important to investigate whether historic evolutionary innovations found in natural systems have properties like the model system studied here. The high probability of finding dual-function sequences in our current data encourages the search for more genotype network intersections and motivates future research on the forecasting of evolutionary innovations.

## Materials and methods

### Library design

For our experiments, we first identified an HDV and a Ligase reference sequence (Fig 1A). For this purpose, we chose sequence variants that were expected to have near wild-type ribozyme fitness and that were 14 mutations apart [24]. We then set out to construct a library of ribozyme sequences that contained all the possible presence–absence combinations of these 14 nucleotide differences. These sequence variants represent all the parsimonious intermediates on the evolutionary trajectories between the two reference sequences. Library construction was accomplished by chemically synthesizing a degenerate DNA oligonucleotide that would serve as a template for in vitro transcription with T7 RNA polymerase. At each position where the Ligase and HDV reference ribozymes differed, the synthesis used equal mixtures of two nucleotide phosphoramidites, generating approximately equal probability of both sequence variants. This creates 2^14^ = 16,384 ribozyme variants. We synthesized two such libraries, one “HDV library” with a 5′-leader sequence that is cleaved by variants with the HDV phenotype and a second “Ligase library” that begins at the 5′-end of the Ligase ribozyme so that variants with the Ligase phenotype could react with a separate substrate oligonucleotide [23]. A common sequence was added to the 3′-end of both libraries to serve as a universal primer binding site for reverse transcription [60]. Oligonucleotides used in this experiment are listed in S1 Table.

### Co-transcriptional cleavage assay

The sample preparation was done entirely in triplicate, yielding 3 biological replicates. The ssDNA ultramer cleavage library used for in vitro transcription of the ribozyme mutants was annealed to the T7-TOP+ primer. 20 picomoles each of DNA template and primer were heated for 5 min at 98°C in 10 μL final volume of custom T7 Mg10 buffer (500 μL 1 M Tris [pH 7.5], 50 μL 1 M DTT, 20 μL 1 M spermidine, 100 μL 1 M MgCl_2_, 330 μL RNase-free water). The template and primer were then diluted 10-fold and cooled to room temperature. 2 μL of template and primer were then transcribed in vitro in a 50 μL reaction with 5 μL T7 Mg10 buffer, 1 μL rNTP (25 mM; New England Biolabs, Ipswich, MA, USA), 1 μL T7 RNA polymerase (200 units; Thermo Fisher Scientific, Waltham, MA, USA) and 41 μL RNase-free water (Ambion, Foster City, CA, USA) at 37°C for 20 min. The transcription was then terminated by adding 15 μL of 50 mM EDTA. Although the total amount of cleaved RNA increases during transcription, the ratio of cleaved to uncleaved remains the same as long as the rate of transcription is constant, which is true for moderately short transcription times before reagents become limited [61]. 20 min was determined to be the optimal time for transcription by transcribing the library at multiple time points and measuring RNA levels using denaturing PAGE (S23A Fig). 20 min was selected as optimal because it was still during linear growth before reaching a plateau. The transcription reaction was then cleaned and concentrated with Direct-zol RNA MicroPrep w/ TRI-Reagent (Zymo Research, Irvine, CA, USA) to 7 μL. The concentration of the RNA sample was then determined using a spectrophotometer (ThermoFisher NanoDrop; Thermo Fisher Scientific), and the samples were normalized to 5 μM. The transcribed and cleaned RNA (5 picomoles) was mixed with 20 picomoles of RT library primer (S1 Table) in a volume of 10 μL and was heated at 72°C for 3 min, then cooled on ice. 4 μL SMARTScribe 5× First-Strand Buffer (Clontech, Takara Bio, Mountain View, CA, USA), 2 μL dNTP (10 mM), 2 μL DTT (20 mM), 2 μL phased template-switching oligo mix (10 μM), 1 μL water, and 1 μL SMARTScribe Reverse Transcriptase (10 units; Clontech) were then added to the RNA template and RT primer. The phased template-switching oligo mix consisted of 4 oligonucleotides that were phased by the addition of 9, 12, 15, or 18 nucleotides (S1 Table). The mixture was then incubated at 42°C for 90 min. The reaction was stopped and the RNA degraded by heating the sample to 72°C for 15 min. The cDNA was then purified using DNA Clean & Concentrator-5 (Zymo Research) and eluted into 7 μL water.

### Ligation assay

The ssDNA ultramer ligation library used for in vitro transcription of the ribozyme mutants was annealed to the T7-TOP+ primer. 20 picomoles each of DNA template and primer were heated for 5 min at 98°C in 10 μL water. The template and primer were then transcribed in vitro in a 30 μL reaction with 12 μL rNTP (25 mM; New England Biolabs), 3 μL MEGAshortscript T7 Reaction Buffer (10×, Thermo Fisher Scientific), and 3 μL MEGAshortscript T7 RNA Polymerase (Thermo Fisher Scientific) at 37°C for 2 hours. The DNA was then degraded using 2 μL TURBO DNase (2 units/μL; Thermo Fisher Scientific) and incubating at 37°C for 15 min. The transcription reaction was then cleaned and concentrated with Direct-zol RNA MicroPrep with TRI-Reagent (Zymo Research) to 7 μL. The concentration of the RNA sample was then determined using a spectrophotometer (ThermoFisher NanoDrop; Thermo Fisher Scientific), and the samples were normalized to 5 μM. To assess the starting abundance of each genotype prior to in vitro selection, a portion of each sample was aliquoted and reverse transcribed using the template-switching protocol identical to what was used for the HDV library. The transcribed and cleaned RNA (25 picomoles) was mixed with 200 mM Tris (pH 7.5) in a volume of 10 μL and heated at 65°C for 2 min and then cooled to room temperature. 500 picomoles of ligation substrate (S1 Table) were then added with 4 μL MgCl_2_ (50 mM) for a total volume of 20 μL. The mixture was then incubated for 2 hours at 37°C. To reverse transcribe the samples, 10 μL of the ligation reaction was heated with 40 picomoles of RT library primer and heated to 72°C for 3 min, then cooled on ice. 4 μL SMARTScribe 5× First-Strand Buffer (Clontech), 2 μL dNTP (10 mM), 2 μL DTT (20 mM), 1 μL water, and 1 μL SMARTScribe Reverse Transcriptase (10 units; Clontech) were then added to the RNA template and RT primer. The mixture was then incubated at 42°C for 90 min. The reaction was stopped and the RNA degraded by heating the sample to 72°C for 15 min. The cDNA was then purified using DNA Clean & Concentrator-5 (Zymo Research) and eluted into 10 μL water. To amplify the cDNA that had performed the ligation reaction, a mix of phased selective-ligation PCR primers were used. The PCR reaction consisted of 1 μL purified cDNA, 12.5 μL KAPA HiFi HotStart ReadyMix (2×; KAPA Biosystems, Wilmington, MA, USA), 2.5 μL selective-ligation primer, 2.5 μL RT primer, and 5 μL water. To prevent bias during the PCR amplification, multiple cycles of PCR were examined using gel electrophoresis, and an appropriate PCR cycle was chosen because it was still in linear growth (S8B Fig). Each PCR cycle consisted of 98°C for 10 s, 63°C for 30 s, and 72°C for 30 s. The PCR cDNA product was then cleaned using DNA Clean & Concentrator-5 (Zymo Research) and eluted in 12 μL water.

### Illumina adapter PCR

In preparation for high-throughput sequencing, Illumina adapter sequences were added to the cDNA using PCR. Each of the 9 samples (3 HDV, 3 ligated, 3 unligated) were each assigned a unique combination of sequencing indices. The PCR reaction consisted of 1 μL purified cDNA, 12.5 μL KAPA HiFi HotStart ReadyMix (2×, KAPA Biosystems), 2.5 μL forward primer, 2.5 μL reverse primer (Illumina Nextera Index Kit; San Diego, CA, USA), and 5 μL water. To prevent bias during the PCR amplification, multiple cycles of PCR were examined using gel electrophoresis, and an appropriate PCR cycle was chosen because it was still in linear growth (S23B Fig). Each PCR cycle consisted of 98°C for 10 s, 63°C for 30 s, and 72°C for 30 s. The PCR cDNA product was then cleaned using DNA Clean & Concentrator-5 (Zymo Research) and eluted in 30 μL water. The final product was then verified using gel electrophoresis.

### High-throughput sequencing

In preparation for high-throughput sequencing, the 3 cleavage replicates, 3 ligated replicates, and 3 unligated replicates, each with unique Illumina adapter barcodes, were pooled and sent to the University of Oregon Genomics and Cell Characterization Core Facility (University of Oregon, Eugene, OR, USA). The samples were sequenced using Illumina NextSeq 500 Single End 150 with 25% PhiX addition. This generated approximately 125 million reads (Cluster PF Yield) across the 9 samples.

### Data analysis

Sequencing data were analyzed using custom Python scripts that are available on GitLab, and all analyses were performed with Python software (Version 3.7.0). For each sequencing read, these scripts identified a universally conserved 3′ handle, determined the reacted state (ligated/unligated or cleaved/uncleaved), and isolated the 14 mutational nucleotides to determine genotype. This process was repeated for each experimental replicate. The uncatalyzed cleavage rate was estimated to be 7 × 10^−7^ min^−1^ [62]. The rates of template-directed, nonenzymatic oligonucleotide ligation were estimated to be 2.4 × 10^−10^ min^−1^ for 2′,5′-linkage and 1.5 × 10^−8^ min^−1^ for 3′,5′-linkage [63,64]. Correlation coefficients were determined between pairs of replicates (S4A Fig). The distribution of HDV and Ligase sequencing read counts were also determined to verify sequencing quality (S6 Fig). The distribution of sequencing read counts for genotypes that were not expected to be in the libraries but were found in our sequencing data was also determined (S7 Fig).

### Ribozyme fitness calculations from sequence data

Fitness values for each genotype were determined from the sequence data. Fitness values for the HDV genotypes were calculated from the fraction of each genotype found in the cleaved form divided by the total reads of that genotype in that sample. These fraction cleaved values were normalized by dividing by the fraction cleaved of a HDV genotype that was in the original intersection paper [24], resulting in the HDV fitness values reported. This resulted in normalizing the data such that the original prototype HDV ribozyme sequence (which is not included in our library) would be equal to 1. The Ligase fitness was determined by the level of enrichment following a round of selection for Ligase activity. The relative abundance of each genotype was determined by dividing the reads corresponding to that genotype by the total number of reads in that replicate sample. The change in abundance was determined by taking the relative abundance of a specific genotype in the sample selected for ligation activity and dividing it by the relative abundance in the initial library before selection. This value was normalized by dividing by the change in abundance for a Ligase genotype that was in the original intersection paper, resulting in the Ligase fitness values reported. This resulted in normalizing the data such that the original prototype Ligase ribozyme sequence (which is not included in our library) would be equal to 1.

### Validation of sequencing-based assays

In order to validate the high-throughput sequencing fitness measurements for HDV (self-cleaving) and Ligase (self-ligation) functions, we developed in vitro biochemical assays. For the HDV activity, we used a gel-based assay (PAGE). Thirteen sequences were ordered as oligos, transcribed, and run on a polyacrylamide gel. Samples were run on 10% denaturing polyacrylamide gel, visualized with GelRed (Biotium, Fremont, CA, USA), and quantified by densitometry. The cleaved and uncleaved products separate in the gel and allow for a calculation of percent cleaved (S5A Fig). Because of the difficulty of getting accurate measurements for Ligase activity using a gel-based assay, we developed a qPCR assay to detect low rates of self-ligation. We cloned several sequences from our library, representing a random sampling of sequences. Each sequence was sequenced to determine the genotype and transcribed to RNA. The RNA was incubated with the Ligase substrate and was allowed to react under identical conditions to the sequencing-based assay. This reaction was reverse transcribed to cDNA and PCR amplified with two primer pairs. One pair was specific to the substrate to measure ligated RNA, and the other pair was specific to the ribozyme and measured total RNA. The ratio of the Cq values of the two PCR signals was used to calculate percent ligated (S5B Fig). We also ordered 4 specific intersection sequences that had very low Ligase activity to validate that low-fitness genotypes are in fact active Ligase ribozymes (S5C Fig). These sequences were analyzed using the qPCR assay, and it is important to note that the ligation activity is dependent on the presence of the substrate. When a control reaction was performed with no substrate, the percent ligated decreased by over 60,000-fold.

### Genotype network and fitness landscape construction

Visualizations of fitness landscapes were constructed using Gephi [65]. Each node represents a unique genotype and edges connecting genotypes represent a single mutation. ForceAtlas 2 was used to approximate genotype repulsion using a Barnes–Hut calculation. The z-axis in the fitness landscape (Fig 3A) was generated using the Network Splitter 3D plugin. Peaks in each fitness landscape were defined as genotypes that were surrounded by mutational neighbors with lower relative fitness. This calculation incorporated the measurement error (delta) between replicates. Ruggedness for each landscape was calculated as the average number of peaks within subgraphs [66]. Each subgraph contains 4 mutational positions with 16 genotypes, and every possible subgraph within each landscape was assessed for peaks. Pairwise epistasis was calculated as *ε* = log_10_ (*W*_AB_ * *W*_0_/*W*_A_ * *W*_B_), where *W*_A_ and *W*_B_ are the fitness of RNA variants with a single mutation, *W*_AB_ is the fitness of the variant with both mutations, and *W*_0_ is the fitness of the background genotype with no mutations [6,67]. Epistasis was calculated for every subgraph of 2 mutational positions containing 4 genotypes. Within each subgraph, there exist equal positive and negative epistatic interactions depending on which genotype is used as the background genotype (W_0_). Therefore, only the magnitude of epistasis within a subgraph is reported.

### Evolutionary simulations

Computational simulations of evolution were accomplished using custom Python scripts (RiboEvolve.py) that model evolution based on the Wright–Fisher approach [35,67]. Simulations were performed on the Boise State R2 computer cluster [68]. A range of population sizes (25, 50, 125, 250, 500, 1,000) and mutation rates (0.0001, 0.01, 0.1, 1.0) were explored (S24 and S25 Figs). For simulations on the Ligase and HDV landscapes, the summit genotype of the opposing landscape was used as the starting genotype. The results show that very high mutation rate (1.0) leads to no adaptation. Very low mutation rates (0.0001) result in no observable evolution under the time frame of our simulations, which are limited by computational expense. We found that mutation rates of 0.01 and 0.1 gave similar results, except that 0.1 reached the summits more quickly, providing less resolution between different adaptation dynamics from different starting genotypes. We therefore prefer the lower mutation rate of 0.01, which we used in our analysis. With this mutation rate, there is only a subtle effect of population size. Therefore, a population size of *N* = 1,000 and mutation rate (*μ*) of 0.01 were used for the remaining simulations.

Simulation started with 1,000 individuals of the same genotype. Every generation (update), a new population of 1,000 genotypes was generated in the following way. First, a parent genotype from the population was selected at random. The fitness of the genotype was compared to a randomly selected value from a fitness range (between 0 and 1). If the genotype fitness was less than the random value, the genotype was not placed in the new generation. If the genotype fitness was greater than or equal to the random value, it was placed in the new generation, with a chance of mutating at a single, randomly chosen nucleotide position. Mutations occurred if a randomly generated number was lower than the mutation rate set at the beginning of the simulation and remained constant (*μ* = 0.01). This process was repeated until 1,000 individuals were placed in the new generation. The simulation then repeated this process for 1,000 generations. We carried out the simulations on the Ligase landscape starting from the 17 genotypes with HDV fitness ≥ 1 and did so for a total of 100 replicates for each genotype (S9 Fig). The 100 replicates for each starting genotype were averaged (Fig 4B), and the initial rate of adaptation and unique genotypes explored for each starting genotype were calculated. For each simulation, simulation rate was determined by subtracting the population fitness at generation = 0 from the population fitness at generation = 200 and dividing this value by 200 generations. Using a cubic spline regression (S10 Fig), we determined the maximum growth rate for the mean fitness of the 100 replicates for each starting genotype (Fig 4E and 4G). We also ran simulations on the HDV landscape starting from the 17 genotypes with the highest Ligase genotypes that also had nonzero HDV fitness. These were repeated for 100 replicates (S15 Fig) and were averaged (Fig 4D). Rate per simulation for the first 200 generations was also calculated for these simulations (Fig 4G). The maximum growth rate for the mean fitness of the 100 replicates for each starting genotype was also determined (S16 Fig).

To understand the role that periods of neutral evolution might play in the evolution of innovations, simulations were performed that introduced a range of neutral evolution intervals (0–1,000 generations). For simulations on the Ligase and HDV landscapes, the summit genotype of the opposing landscape was used as the starting genotype. Following generations of neutral evolution, selection pressure was immediately applied for the remainder of the 1,000 generations. This was repeated in each scenario for 100 replicates (S17 and S18 Figs). Lastly, simulations were conducted on an HDV–Ligase coselection landscape that allows selection to act upon both functions simultaneously. For this model, the fitness was calculated as *W*_*HDV*_ * *β*_*HDV*_ + *W*_*Ligase*_ * *β*_*Ligase*_, where *W* indicates the fitness of that function and *β* indicates a weighting parameter that can be adjusted (S21 and S22 Figs). Otherwise, simulations were the same as above.

## Supporting information

S1 Fig Overview of high-throughput ribozyme functional assays. Detailed approach used to assess the relative fitness of each of the 16,384 genotypes for two functions: self-cleavage (HDV) and self-ligation (Ligase). Note that in order to assess the preselection frequency of genotypes in the self-ligation assay, a portion of the RNA library is processed using the protocol used in the self-cleavage assay (dotted line). HDV, Hepatitis Delta Virus.
(PNG)

S2 Fig Overlay of HDV and Ligase genotype networks with varying fitness cutoffs. Each plot indicates the overlay of the two networks with all genotypes with fitness values below the cutoff removed. The size of the node indicates relative fitness, and nodes are colored based on their dominant activity (red = HDV, blue = Ligase). Below each plot is the range of mutations needed to go from one network to the other. The distribution of the number of mutations required is displayed for each fitness cutoff in Fig 2A. HDV, Hepatitis Delta Virus.
(PNG)

S3 Fig Correlation between mutational distance from the opposing genotype network and active connections. The mutational distance was calculated from varying the fitness cutoff for each landscape as in Fig 2A. As the mutational distance for genotypes shifted, we calculated the number of active connections on the initial function landscape, as well as connection to the new function landscape. This was repeated using the HDV and the Ligase function as the initial function. Data and Python scripts for connection calculations can be found on GitLab. HDV, Hepatitis Delta Virus.
(PNG)

S4 Fig High-throughput sequencing results for HDV and Ligase. (A) Correlation of total HDV and Ligase reads for each of the 3 replicates. Each figure consists of all 16,384 genotypes presented in this study. Each data point represents the frequency that a specific sequence was observed in a particular replicate (y-axis) versus another replicate (x-axis). Sequence kernel density estimation is also reported from each replicate in the jointplot (Seaborn python package). The number of reads on the x- and y-axis are log10 transformed. Pearson (R^2^) and Spearman (ρ) correlation is reported for each correlation. Data and Python scripts for correlations can be found on GitLab. (B) Error rates calculated from base miscalls in the PhiX reference genome. Error rate (y-axis) is shown for the 14 positions (x-axis) where our genotypes are defined. Each position is read in 4 different sequencing cycles, and error rates are reported as the average error rate of these 4 cycles. Dashed blue line indicates the average error rate across all 14 mutational positions. Error rates are calculated by aligning each PhiX sequence read in our data to the reference PhiX genome and counting mismatches at each sequence cycle. Data and Python scripts for the calculation of sequencing error rates can be found on GitLab. HDV, Hepatitis Delta Virus; PhiX, phi X bacteriophage genome control.
(PNG)

S5 Fig Validation of high-throughput-sequencing–based assays. (A) Correlation of fitness values for 13 unique ribozyme genotypes assessed by high-throughput sequencing and gel-based assay (PAGE). Both methods assessed the fraction cleaved (fitness) of each genotype. Pearson and Spearman correlations are reported. Data and Python scripts for correlation can be found on GitLab. (B) Correlation of fitness values for 19 unique ribozyme genotypes assessed by high-throughput sequencing and qPCR assays. Data and Python scripts for correlation can be found on GitLab. (C) qPCR measurements of 4 low-fitness intersection sequences. The 4 intersection sequences were determined to have low fitness (<0.03) by the high-throughput sequencing assay. qPCR, quantitative Polymerase Chain Reaction.
(PNG)

S6 Fig Distribution of expected sequencing read counts. (A) Histograms indicating the average read counts for each individual genotype in the designed HDV library for all 3 replicates. The mean read count for each genotype across HDV replicates was 369. Dashed line indicates the mean within a replicate. Data and Python scripts for the calculation and plotting of the read counts can be found on GitLab. (B) Histograms indicating the average read counts for each individual genotype in the designed Ligase library for all 3 replicates. The mean read count for each genotype across Ligase replicates was 230. Dashed line indicates the mean within a replicate. Data and Python scripts for the calculation and plotting of the read counts can be found on GitLab. HDV, Hepatitis Delta Virus.
(PNG)

S7 Fig Distribution of unexpected sequencing read counts. (A) Histograms indicating the average read counts for each unexpected genotype found in the HDV sequencing samples. Unexpected genotypes were those that were not expected in our sequencing library but were found in the sequencing data. Dashed line indicates the mean within a replicate. Data and Python scripts for the calculation and plotting of the read counts can be found on GitLab. (B) Histograms indicating the average read counts for each unexpected genotype found in the Ligase sequencing samples. Dashed line indicates the mean within a replicate. Data and Python scripts for the calculation and plotting of the read counts can be found on GitLab. HDV, Hepatitis Delta Virus.
(PNG)

S8 Fig Evaluation of Ligase fitness measurements. (A) Correlation of Ligase fitness and the relative frequency preselection. Each figure consists of all 16,384 genotypes presented in this study. Each data point represents the frequency that a specific sequence was observed in a particular replicate preselection (y-axis) versus the average calculated Ligase “fitness.” Data and Python scripts for correlation can be found on GitLab. (B) Correlation of Ligase fitness and relative rank preselection. Each data point represents the relative rank (1–16,384) based on the average number of reads per replicate preselection (y-axis) versus the average calculated Ligase “fitness.” Data and Python scripts for correlation can be found on GitLab. (C) Ligase fitness landscape depicted as a function of estimated “ligation rate.” Ligation rate was estimated using a reference genotype that was found in our data set and the original intersection study [24]. The ligation rates for template-directed, nonenzymatic oligonucleotide ligation are estimated as 2.4 × 10^−10^ min^−1^ for 2′–5′ ligation [64] and 1.5 × 10^−8^ min^−1^ for 3′–5′ ligation [63]. Data and Python scripts for the calculation and plotting of relative ligation rate can be found on GitLab.
(PNG)

S9 Fig Rate of adaptation for populations starting from different genotypes on the Ligase landscape. Each trace shows the increase in population fitness over generation time for a single simulation of 1,000 individuals. Each plot shows 100 simulations starting from the same genotype. All starting genotypes has HDV fitness ≥ 1. The letter above each subplot indicates the starting point from the network, as shown in Fig 3A. Letters were assigned alphabetically based on highest to lowest HDV fitness, and genotype a represents the genotype with the highest measured HDV fitness. The graphs are ordered from fastest to slowest initial rates (Fig 4D). Data and Python scripts for evolutionary simulations can be found on GitLab. HDV, Hepatitis Delta Virus.
(PNG)

S10 Fig Regression analysis of average rates of fitness optimization on the Ligase landscape. Regression was performed for each starting genotype (REF–p). Solid points are the average of 100 replicates of simulated evolution and correspond to the data from Fig 4B. Dashed black lines are the fitted regression line. The maximum growth rate (*μ*) derived from the regression is reported on each plot. Data and Python scripts for the regression analysis can be found on GitLab. REF, reference.
(PNG)

S11 Fig Relationship between maximum growth rate and mutational distance. Mutational distance is calculated as the number of mutations between the summit genotype and a given starting genotype for the evolutionary simulations (Fig 4). Data and Python scripts for the relationship can be found on GitLab.
(PNG)

S12 Fig Trajectories away from stasis genotypes. (A) Each line leads from the stasis genotype (mutations = 0) to 1 and 2 mutations away. All 69 stasis genotypes (peaks) in the Ligase fitness landscape are depicted. The number on each graph represents the number of 2-mutation pathways to higher fitness from each stasis genotype. The yellow box indicates the genotype with the highest measured Ligase fitness. Data and Python scripts for calculation and plotting of mutational pathways can be found on GitLab. (B) The distribution of 2-mutation pathways to higher-fitness genotypes from each stasis genotypes in the Ligase landscape. The dotted vertical line indicates the mean of the distribution.
(PNG)

S13 Fig Characterization of stasis genotype I. Stasis genotype I from Fig 4F is depicted in the center with each of the 2 mutation trajectories. None of the 182 2-mutation trajectories lead to higher fitness than the stasis genotype (mutation = 0). The pathways 2 mutations from each of the 14 genotypes that are a single mutation away from the stasis genotype are individually depicted. In total, 42 out of a possible 2,184 3-mutation trajectories yield a higher fitness than the initial stasis genotype (dashed line). Data and Python scripts for calculation and plotting of mutational pathways can be found on GitLab.
(PNG)

S14 Fig Rates of adaptation are not altered by fitness precision of low-fitness genotypes. Final fitness of 100 replicate simulations on the original Ligase landscape (red) or a landscape where the lowest-fitness genotypes were converted to fitness = 0 (gray), if fitness was less than 0.005 (7,015 genotypes converted to fitness = 0). Simulations were carried out as in Fig 4 of the main text, with 1,000 individuals and 1,000 generations each. Data and Python scripts for evolutionary simulations can be found on GitLab.
(PNG)

S15 Fig Rate of adaptation for populations starting from different genotypes on the HDV landscape. Each trace shows the increase in population fitness over generation time for a single simulation of 1,000 individuals. Each plot shows 100 simulations starting from the same genotype. The letter above each subplot indicates the starting point from the network, as shown in Fig 3A. Letters were assigned alphabetically based on highest to lowest Ligase fitness, and genotype A represents the genotype with the highest measured Ligase fitness. The graphs are ordered from fastest to slowest initial rates (Fig 4E). Data and Python scripts for evolutionary simulations can be found on GitLab.
(PNG)

S16 Fig Regression analysis of average rates of fitness optimization on the HDV landscape. Regression was performed for each starting genotype (A–Q). Solid points are the average of 100 replicates of simulated evolution and correspond to the data from Fig 4D. Dashed black lines are the fitted regression line. The maximum growth rate (*μ*) derived from the regression is reported on each plot. Data and Python scripts for the regression analysis can be found on GitLab. HDV, Hepatitis Delta Virus.
(PNG)

S17 Fig Rate of adaptation for populations following different lengths of neutral evolution on the Ligase landscape. Each trace shows the increase in population fitness over generation time for a single simulation of 1,000 individuals. Each plot shows 100 simulations starting from the summit genotype of the HDV landscape. The number above each subplot indicates the number of generations of neutral evolution before selection was applied as shown in Fig 5. Data and Python scripts for evolutionary simulations can be found on GitLab. HDV, Hepatitis Delta Virus.
(PNG)

S18 Fig Rate of adaptation for populations following different lengths of neutral evolution on the HDV landscape. Each trace shows the increase in population fitness over generation time for a single simulation of 1,000 individuals. Each plot shows 100 simulations starting from the summit genotype of the Ligase landscape. The number above each subplot indicates the number of generations of neutral evolution before selection was applied as shown in Fig 5. Data and Python scripts for evolutionary simulations can be found on GitLab. HDV, Hepatitis Delta Virus.
(PNG)

S19 Fig Regression analysis of average rates of fitness optimization on the Ligase landscape following neutral evolution. Cubic spline regression was performed for the range of periods of neutral evolution (0–1,000). Solid points are the average of 100 replicates of simulated evolution and correspond to the data from Fig 5A. Dashed black lines are the fitted regression line. The maximum growth rate (*μ*) derived from the regression is reported on each plot. Data and Python scripts for the regression analysis can be found on GitLab.
(PNG)

S20 Fig Regression analysis of average rates of fitness optimization on the HDV landscape following neutral evolution. Cubic spline regression was performed for the range of periods of neutral evolution (0–1,000). Solid points are the average of 100 replicates of simulated evolution and correspond to the data from Fig 5B. Dashed black lines are the fitted regression line. The maximum growth rate (*μ*) derived from the regression is reported on each plot. Data and Python scripts for the regression analysis can be found on GitLab. HDV, Hepatitis Delta Virus.
(PNG)

S21 Fig Evolutionary simulations on the HDV–Ligase coselect fitness landscapes. (A) The starting population that was randomly selected from the 3,432 genotypes that are 7 mutations from HDV reference and Ligase reference. (B) Average rates of evolutionary adaptation on the HDV–Ligase coselect fitness landscapes with varying weighted parameters (β) for each function. Line indicates the average of 100 replicates (S22 Fig). Total population fitness indicates the fitness resulting from the following equation, *W*_*HDV*_ * *β*_*HDV*_ + *W*_*Ligase*_ * *β*_*Ligase*_, where *W* indicates the fitness of that function and *β* indicates a weighting parameter that was adjusted. The HDV and Ligase population is also plotted independently to indicate which function is the dominant contributor to the total fitness. Line color indicates the weighting parameters used in the simulation as indicated in the top-right inset. Data and Python scripts for evolutionary simulations can be found on GitLab. HDV, Hepatitis Delta Virus.
(PNG)

S22 Fig Individual traces of evolutionary adaptation on HDV–Ligase coselect fitness landscapes. (A–C) Left plot indicates the architecture of the fitness landscape for each combination of weighting parameters (β). Total fitness (calculated as *W*_*HDV*_ * *β*_*HDV*_ + *W*_*Ligase*_ * *β*_*Ligase*_), HDV fitness, and Ligase fitness are shown for each individual simulation replicate. Color of lines correspond to the weighting parameters discussed in S21 Fig. Data and Python scripts for evolutionary simulations can be found on GitLab. HDV, Hepatitis Delta Virus.
(PNG)

S23 Fig Time courses for sample optimization and validation of sequencing fitness values. (A) Time-course transcription for total RNA yield using the developed co-transcriptional cleavage assay. Data points indicate the mean RNA yield of 5 replicates. Error bars are standard error of the mean. Samples were run on 10% denaturing polyacrylamide gel, visualized with GelRed (Biotium), and quantified by densitometry. The time chosen as optimal (20 min) is indicated with a box. (B) Time-course PCR was performed for the selective-ligation PCR and each Illumina adapter PCR for each replicate (blue, green, red). Samples were run on 2% agarose gel, visualized with GelRed (Biotium), and quantified by densitometry. The black box indicates the PCR cycle that was determined to be optimal for each PCR reaction.
(PNG)

S24 Fig Rate of adaptation for populations using a range of population sizes and mutation rates. Average rates of evolutionary adaptation of HDV and Ligase activity starting from the summit genotype of the opposing landscape. Trace color indicates the varying population sizes (25–1,000) as indicated in the legend. Each plot indicates a different mutation rate (0.0001–1.0). Each trace shows the mean fitness of 100 simulations as a function of time (generation). The vertical dashed line marks generation 200. Data and Python scripts for evolutionary simulations can be found on GitLab. HDV, Hepatitis Delta Virus.
(PNG)

S25 Fig Initial rates of adaptation for populations using a range of population sizes and mutation. Distributions of initial rates of adaptation on the Ligase and HDV landscape. Initial rate is determined as the rate of population increase for the first 200 generations. Each violin plot represents the distribution of 100 simulations using the same population size and mutation rate. Plot color indicates the varying population sizes (25–1,000) as indicated in the legend. Mutation rate (0.0001–1.0) is indicated on the x-axis. Data and Python scripts for evolutionary simulations can be found on GitLab. HDV, Hepatitis Delta Virus.
(PNG)

S1 Table Oligonucleotides used in this study. (PNG)

S2 Table Starting genotypes used in evolution simulations. Genotypes are represented by the unique combination of nucleotides in the 14 variable positions of the library. Starting point letters correspond to Fig 3A. HDV and Ligase fitness are colored with bar graphs indicating the relative fitness. HDV, Hepatitis Delta Virus.
(PNG)

S1 Data HDV–Ligase fitness measurements from high-throughput sequencing assays. The fitness measurements for each of the 16,384 unique genotypes presented in this study. The genotypes are displayed as the 14 mutational positions. HDV and Ligase fitness values are colored according to the relative fitness for easier interpretation. Delta values were calculated as the standard error between the 3 sequencing replicates for each function. HDV, Hepatitis Delta Virus.
(XLSX)

S1 Movie Aerial overview of the HDV–Ligase fitness landscape. Overview of the empirical HDV–Ligase fitness landscape presented in Fig 3A. Each node represents an individual genotype, and edges connect nodes that differ by a single nucleotide. The size and height of each node indicates the relative genotype fitness (HDV = red, Ligase = blue). Data and Python scripts for evolutionary simulations can be found on GitLab. HDV, Hepatitis Delta Virus.
(AVI)

S2 Movie Simulated evolution on Ligase landscape starting at genotype k. White nodes are genotypes in the fitness landscape. Genotypes are connected by light blue edges if they differ by a single nucleotide change. The size of the blue circles depicts the relative proportion of the simulated population at that genotype. The y-axis is relative Ligase fitness. The x-axis is number of nucleotide differences from the HDV reference sequence (mutational distance). The number above the graph represents the generation number. The population average is also depicted with the number of generations on the x-axis and mean population fitness on the y-axis. Lastly, the population diversity at a given generation is plotted as a function of generational time. Population diversity indicates the number of unique genotypes present in the population. Data and Python scripts for evolutionary simulations can be found on GitLab. HDV, Hepatitis Delta Virus.
(MP4)

S3 Movie Simulated evolution on Ligase landscape starting at genotype a. White nodes are genotypes in the fitness landscape. Genotypes are connected by light blue edges if they differ by a single nucleotide change. The size of the blue circles depicts the relative proportion of the simulated population at that genotype. The y-axis is relative Ligase fitness. The x-axis is number of nucleotide differences from the HDV reference sequence (mutational distance). The number above the graph represents the generation number. The population average is also depicted with the number of generations on the x-axis and mean population fitness on the y-axis. Lastly, the population diversity at a given generation is plotted as a function of generational time. Population diversity indicates the number of unique genotypes present in the population. Data and Python scripts for evolutionary simulations can be found on GitLab. HDV, Hepatitis Delta Virus.
(MP4)

S4 Movie Simulated evolution on Ligase landscape starting at genotype b. White nodes are genotypes in the fitness landscape. Genotypes are connected by light blue edges if they differ by a single nucleotide change. The size of the blue circles depicts the relative proportion of the simulated population at that genotype. The y-axis is relative Ligase fitness. The x-axis is number of nucleotide differences from the HDV reference sequence (mutational distance). The number above the graph represents the generation number. The population average is also depicted with the number of generations on the x-axis and mean population fitness on the y-axis. Lastly, the population diversity at a given generation is plotted as a function of generational time. Population diversity indicates the number of unique genotypes present in the population. Data and Python scripts for evolutionary simulations can be found on GitLab. HDV, Hepatitis Delta Virus.
(MP4)

S5 Movie Simulated evolution on Ligase landscape starting at genotype m. White nodes are genotypes in the fitness landscape. Genotypes are connected by light blue edges if they differ by a single nucleotide change. The size of the blue circles depicts the relative proportion of the simulated population at that genotype. The y-axis is relative Ligase fitness. The x-axis is number of nucleotide differences from the HDV reference sequence (mutational distance). The number above the graph represents the generation number. The population average is also depicted with the number of generations on the x-axis and mean population fitness on the y-axis. Lastly, the population diversity at a given generation is plotted as a function of generational time. Population diversity indicates the number of unique genotypes present in the population. Data and Python scripts for evolutionary simulations can be found on GitLab. HDV, Hepatitis Delta Virus.
(MP4)

S6 Movie Simulated evolution on HDV landscape starting at genotype A. White nodes are genotypes in the fitness landscape. Genotypes are connected by light red edges if they differ by a single nucleotide change. The size of the red circles depicts the relative proportion of the simulated population at that genotype. The y-axis is relative HDV fitness. The x-axis is number of nucleotide differences from the HDV reference sequence (mutational distance). The number above the graph represents the generation number. The population average is also depicted with the number of generations on the x-axis and mean population fitness on the y-axis. Lastly, the population diversity at a given generation is plotted as a function of generational time. Population diversity indicates the number of unique genotypes present in the population. Data and Python scripts for evolutionary simulations can be found on GitLab. HDV, Hepatitis Delta Virus.
(MP4)

S7 Movie Simulated evolution on HDV landscape following 300 generations of neutral evolution. Simulations started at genotype A. White nodes are genotypes in the fitness landscape. Genotypes are connected by light red edges if they differ by a single nucleotide change. The size of the blue circles depicts the relative proportion of the simulated population at that genotype. The y-axis is relative Ligase fitness. The x-axis is number of nucleotide differences from the HDV reference sequence (mutational distance). The number above the graph represents the generation number. The population average is also depicted with the number of generations on the x-axis and mean population fitness on the y-axis. Lastly, the population diversity at a given generation is plotted as a function of generational time. Population diversity indicates the number of unique genotypes present in the population. Data and Python scripts for evolutionary simulations can be found on GitLab. HDV, Hepatitis Delta Virus.
(MP4)

S8 Movie Simulated evolution on HDV landscape following 600 generations of neutral evolution. Simulations started at genotype A. White nodes are genotypes in the fitness landscape. Genotypes are connected by light red edges if they differ by a single nucleotide change. The size of the blue circles depicts the relative proportion of the simulated population at that genotype. The y-axis is relative Ligase fitness. The x-axis is number of nucleotide differences from the HDV reference sequence (mutational distance). The number above the graph represents the generation number. The population average is also depicted with the number of generations on the x-axis and mean population fitness on the y-axis. Lastly, the population diversity at a given generation is plotted as a function of generational time. Population diversity indicates the number of unique genotypes present in the population. Data and Python scripts for evolutionary simulations can be found on GitLab. HDV, Hepatitis Delta Virus.
(MP4)

S9 Movie Simulated evolution on HDV landscape following 900 generations of neutral evolution. Simulations started at genotype A. White nodes are genotypes in the fitness landscape. Genotypes are connected by light red edges if they differ by a single nucleotide change. The size of the blue circles depicts the relative proportion of the simulated population at that genotype. The y-axis is relative Ligase fitness. The x-axis is number of nucleotide differences from the HDV reference sequence (mutational distance). The number above the graph represents the generation number. The population average is also depicted with the number of generations on the x-axis and mean population fitness on the y-axis. Lastly, the population diversity at a given generation is plotted as a function of generational time. Population diversity indicates the number of unique genotypes present in the population. Data and Python scripts for evolutionary simulations can be found on GitLab. HDV, Hepatitis Delta Virus.
(MP4)

S10 Movie Simulated evolution on Ligase landscape following 300 generations of neutral evolution. Simulations started at genotype a. White nodes are genotypes in the fitness landscape. Genotypes are connected by light blue edges if they differ by a single nucleotide change. The size of the blue circles depicts the relative proportion of the simulated population at that genotype. The y-axis is relative Ligase fitness. The x-axis is number of nucleotide differences from the HDV reference sequence (mutational distance). The number above the graph represents the generation number. The population average is also depicted with the number of generations on the x-axis and mean population fitness on the y-axis. Lastly, the population diversity at a given generation is plotted as a function of generational time. Population diversity indicates the number of unique genotypes present in the population. Data and Python scripts for evolutionary simulations can be found on GitLab. HDV, Hepatitis Delta Virus.
(MP4)

## Abbreviations

HDV: Hepatitis Delta Virus
