# Supplementary figures and images for: Genotype network intersections promote evolutionary innovation
Source: PLoS Biol. 2019 May 28;17(5):e3000300. doi: 10.1371/journal.pbio.3000300 (PMC6555535; doi:10.1371/journal.pbio.3000300)

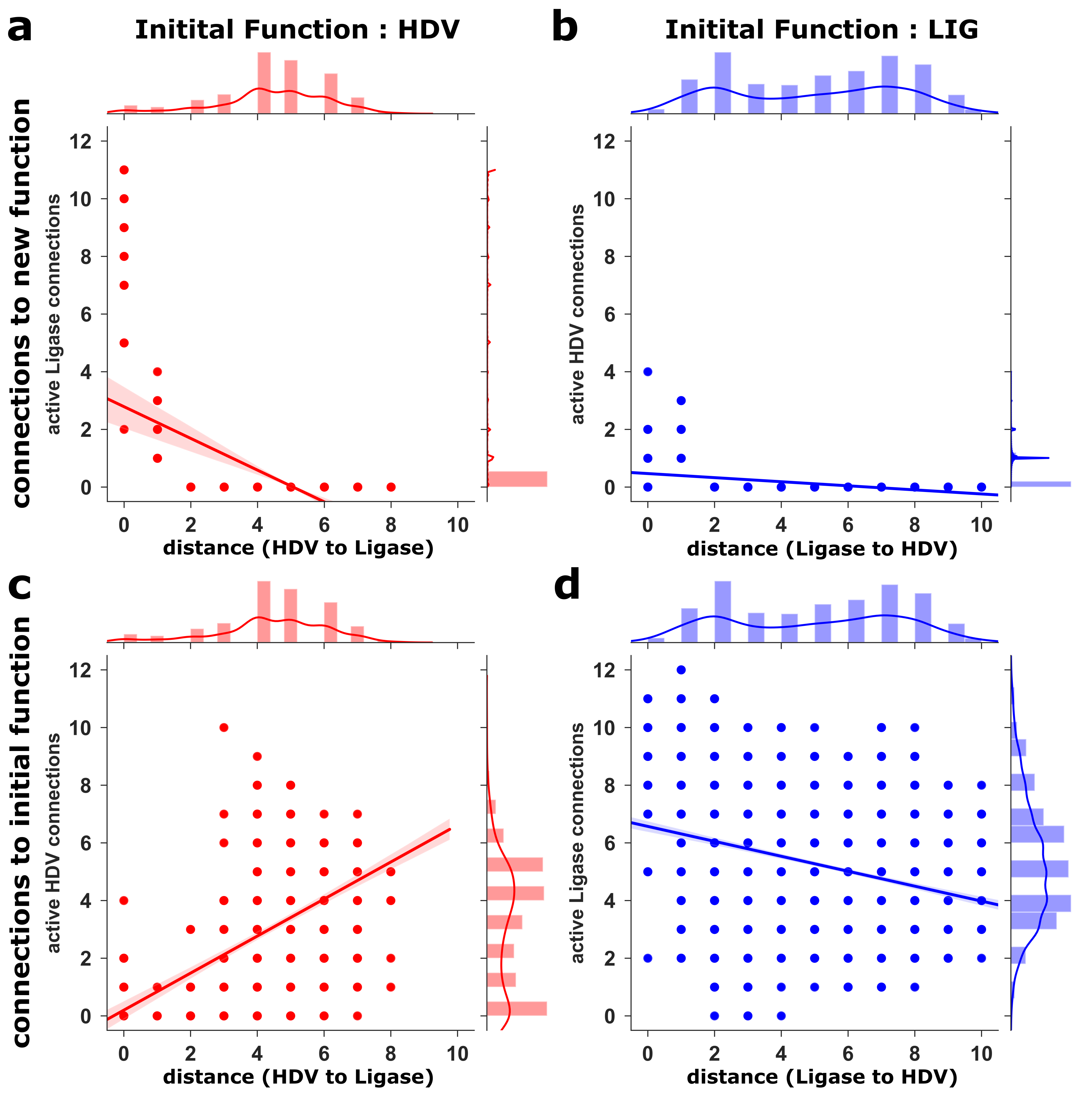

Supplement: S3 Fig — The mutational distance was calculated from varying the fitness cutoff for each landscape as in Fig 2A. As the mutational distance for genotypes shifted, we calculated the number of active connections on the initial function landscape, as well as connection to the new function landscape. This was repeated using the HDV and the Ligase function as the initial function. Data and Python scripts for connection calculations can be found on GitLab. HDV, Hepatitis Delta Virus. (PNG) [file pbio.3000300.s003.png]

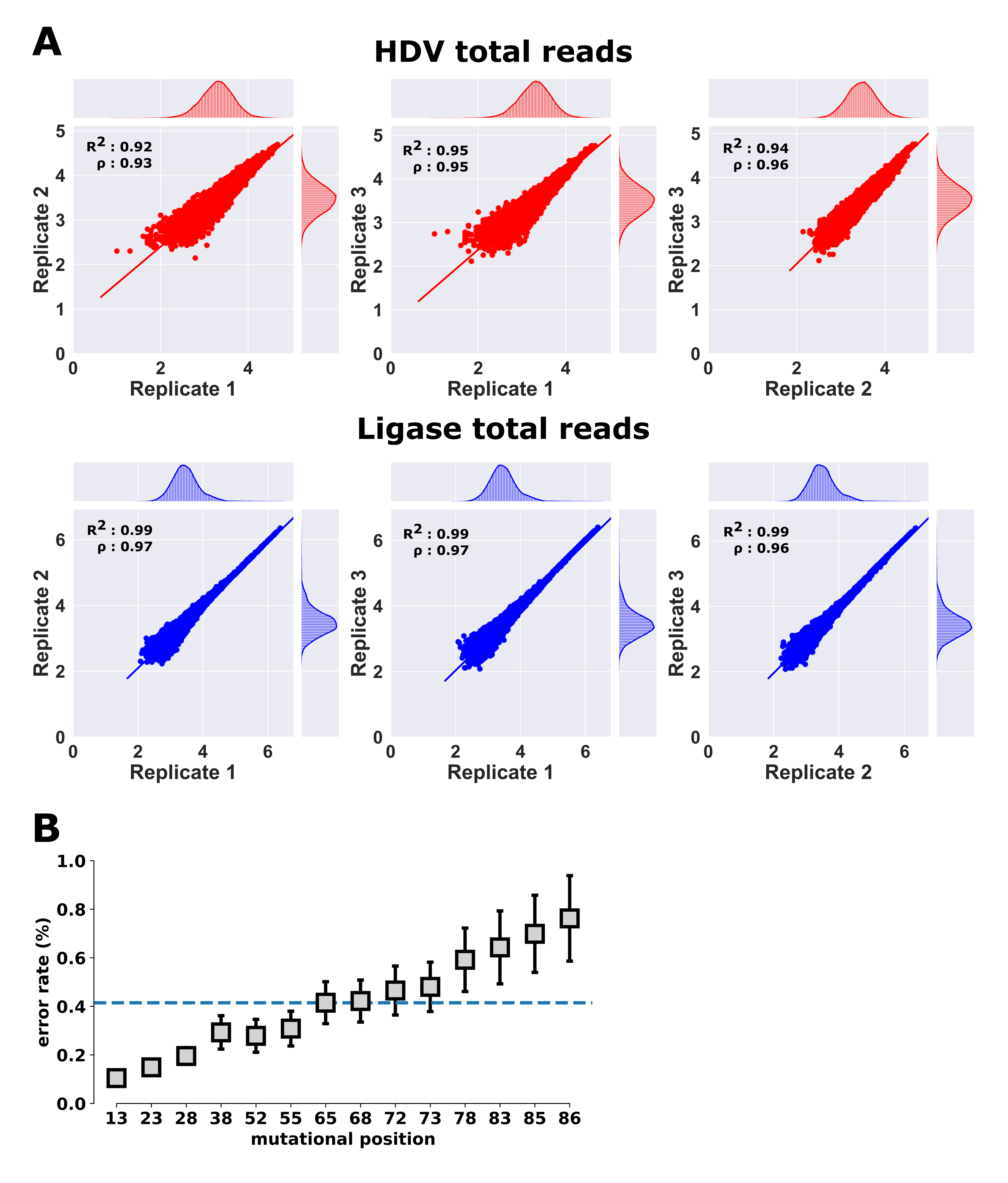

Supplement: S4 Fig — (A) Correlation of total HDV and Ligase reads for each of the 3 replicates. Each figure consists of all 16,384 genotypes presented in this study. Each data point represents the frequency that a specific sequence was observed in a particular replicate (y-axis) versus another replicate (x-axis). Sequence kernel density estimation is also reported from each replicate in the jointplot (Seaborn python package). The number of reads on the x- and y-axis are log10 transformed. Pearson (R2) and Spearman (ρ) correlation is reported for each correlation. Data and Python scripts for correlations can be found on GitLab. (B) Error rates calculated from base miscalls in the PhiX reference genome. Error rate (y-axis) is shown for the 14 positions (x-axis) where our genotypes are defined. Each position is read in 4 different sequencing cycles, and error rates are reported as the average error rate of these 4 cycles. Dashed blue line indicates the average error rate across all 14 mutational positions. Error rates are calculated by aligning each PhiX sequence read in our data to the reference PhiX genome and counting mismatches at each sequence cycle. Data and Python scripts for the calculation of sequencing error rates can be found on GitLab. HDV, Hepatitis Delta Virus; PhiX, phi X bacteriophage genome control. (PNG) [file pbio.3000300.s004.png]

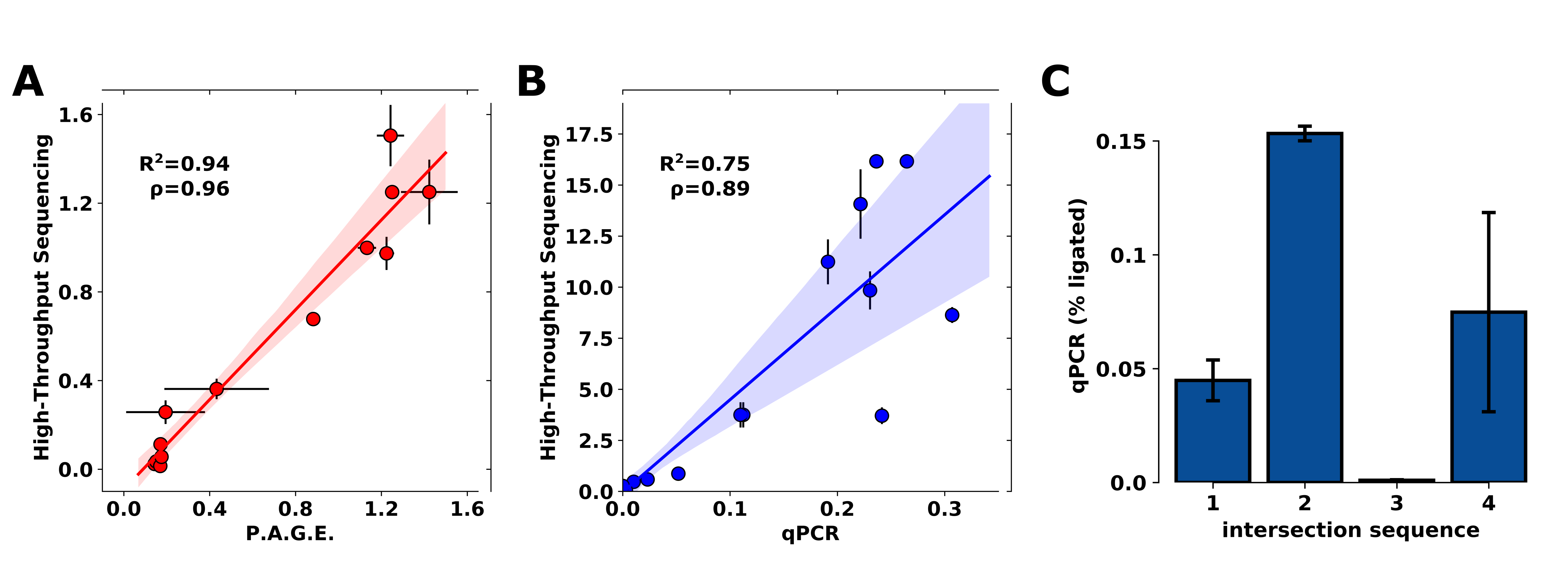

Supplement: S5 Fig — (A) Correlation of fitness values for 13 unique ribozyme genotypes assessed by high-throughput sequencing and gel-based assay (PAGE). Both methods assessed the fraction cleaved (fitness) of each genotype. Pearson and Spearman correlations are reported. Data and Python scripts for correlation can be found on GitLab. (B) Correlation of fitness values for 19 unique ribozyme genotypes assessed by high-throughput sequencing and qPCR assays. Data and Python scripts for correlation can be found on GitLab. (C) qPCR measurements of 4 low-fitness intersection sequences. The 4 intersection sequences were determined to have low fitness (<0.03) by the high-throughput sequencing assay. qPCR, quantitative Polymerase Chain Reaction. (PNG) [file pbio.3000300.s005.png]

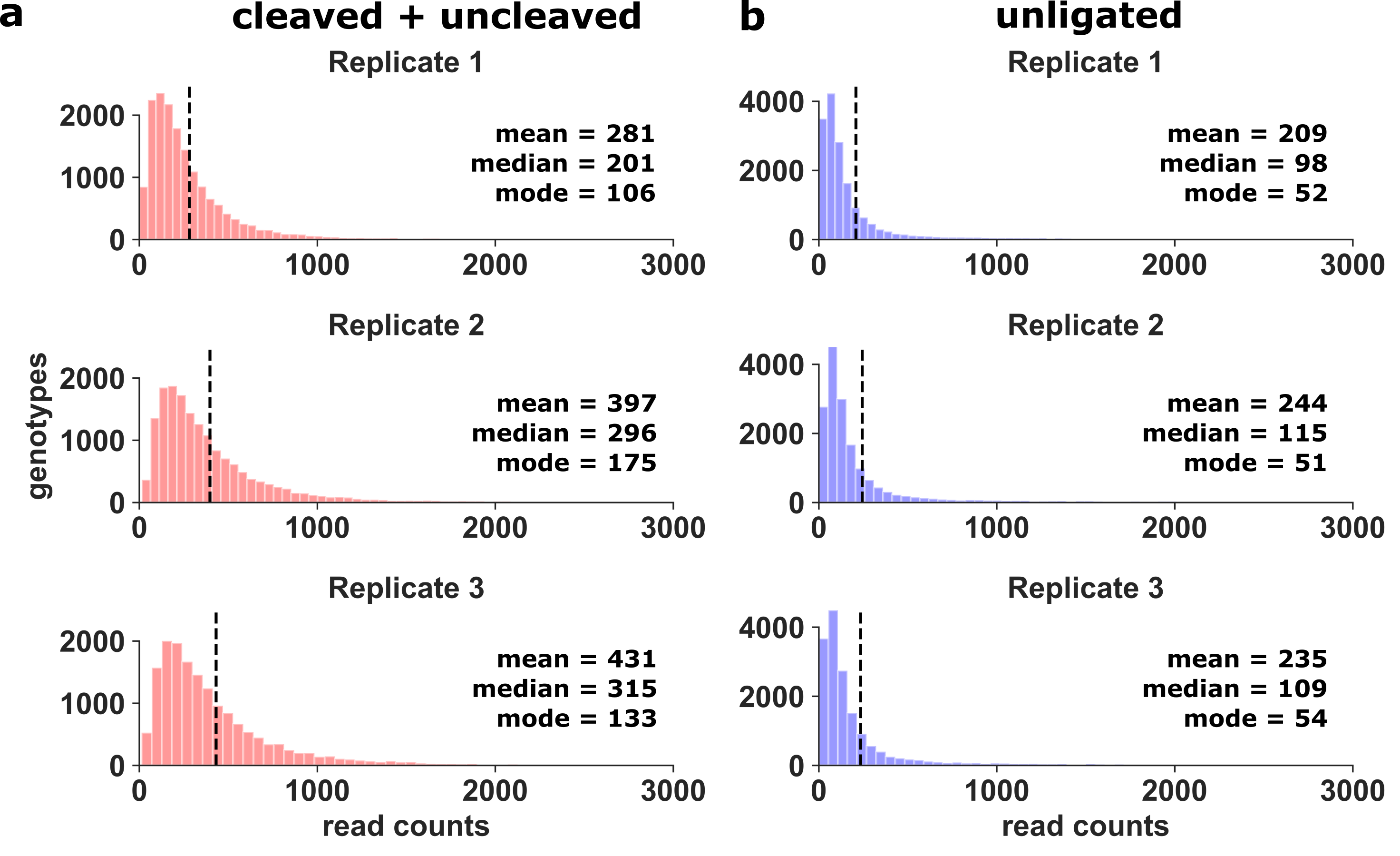

Supplement: S6 Fig — (A) Histograms indicating the average read counts for each individual genotype in the designed HDV library for all 3 replicates. The mean read count for each genotype across HDV replicates was 369. Dashed line indicates the mean within a replicate. Data and Python scripts for the calculation and plotting of the read counts can be found on GitLab. (B) Histograms indicating the average read counts for each individual genotype in the designed Ligase library for all 3 replicates. The mean read count for each genotype across Ligase replicates was 230. Dashed line indicates the mean within a replicate. Data and Python scripts for the calculation and plotting of the read counts can be found on GitLab. HDV, Hepatitis Delta Virus. (PNG) [file pbio.3000300.s006.png]

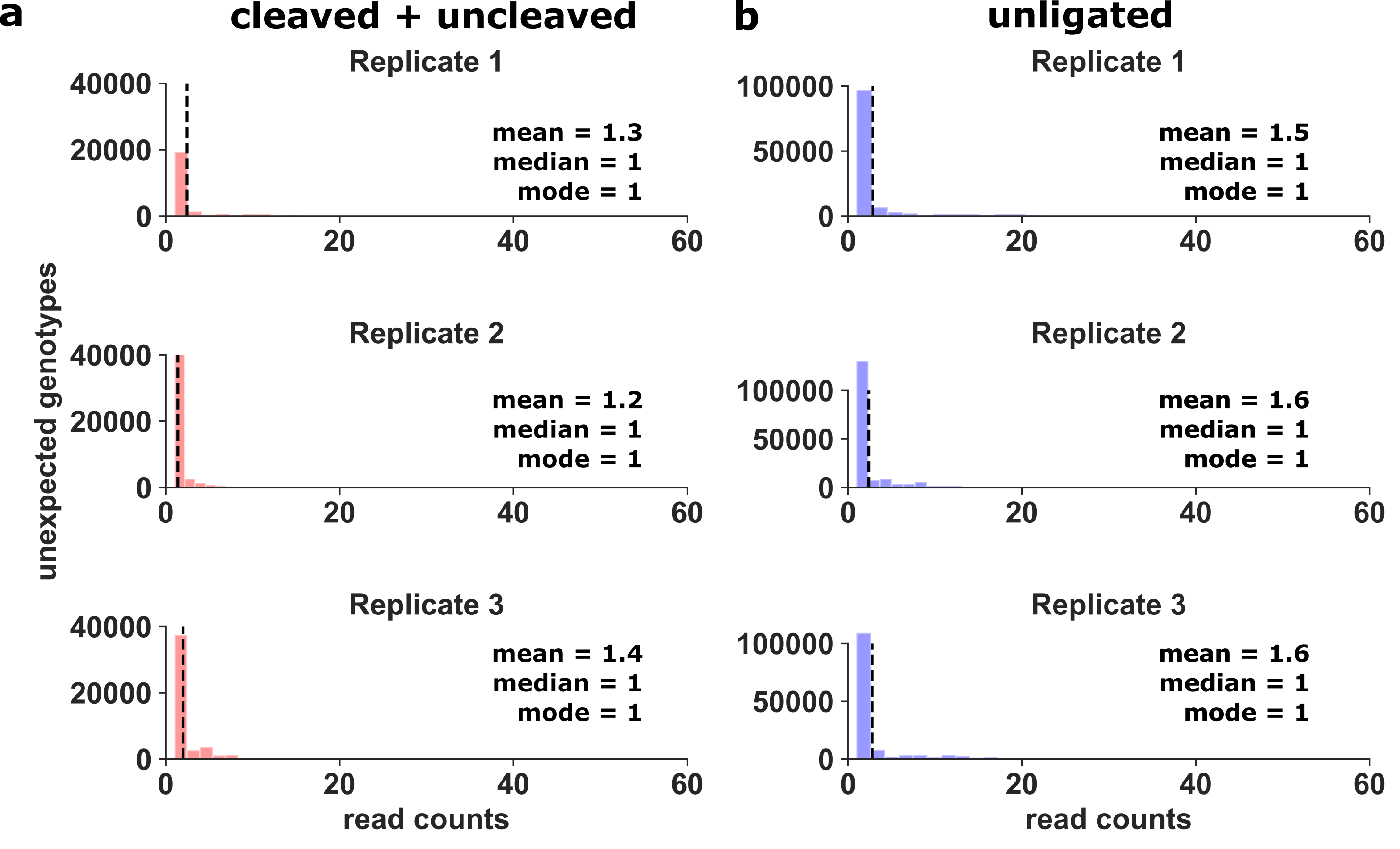

Supplement: S7 Fig — (A) Histograms indicating the average read counts for each unexpected genotype found in the HDV sequencing samples. Unexpected genotypes were those that were not expected in our sequencing library but were found in the sequencing data. Dashed line indicates the mean within a replicate. Data and Python scripts for the calculation and plotting of the read counts can be found on GitLab. (B) Histograms indicating the average read counts for each unexpected genotype found in the Ligase sequencing samples. Dashed line indicates the mean within a replicate. Data and Python scripts for the calculation and plotting of the read counts can be found on GitLab. HDV, Hepatitis Delta Virus. (PNG) [file pbio.3000300.s007.png]

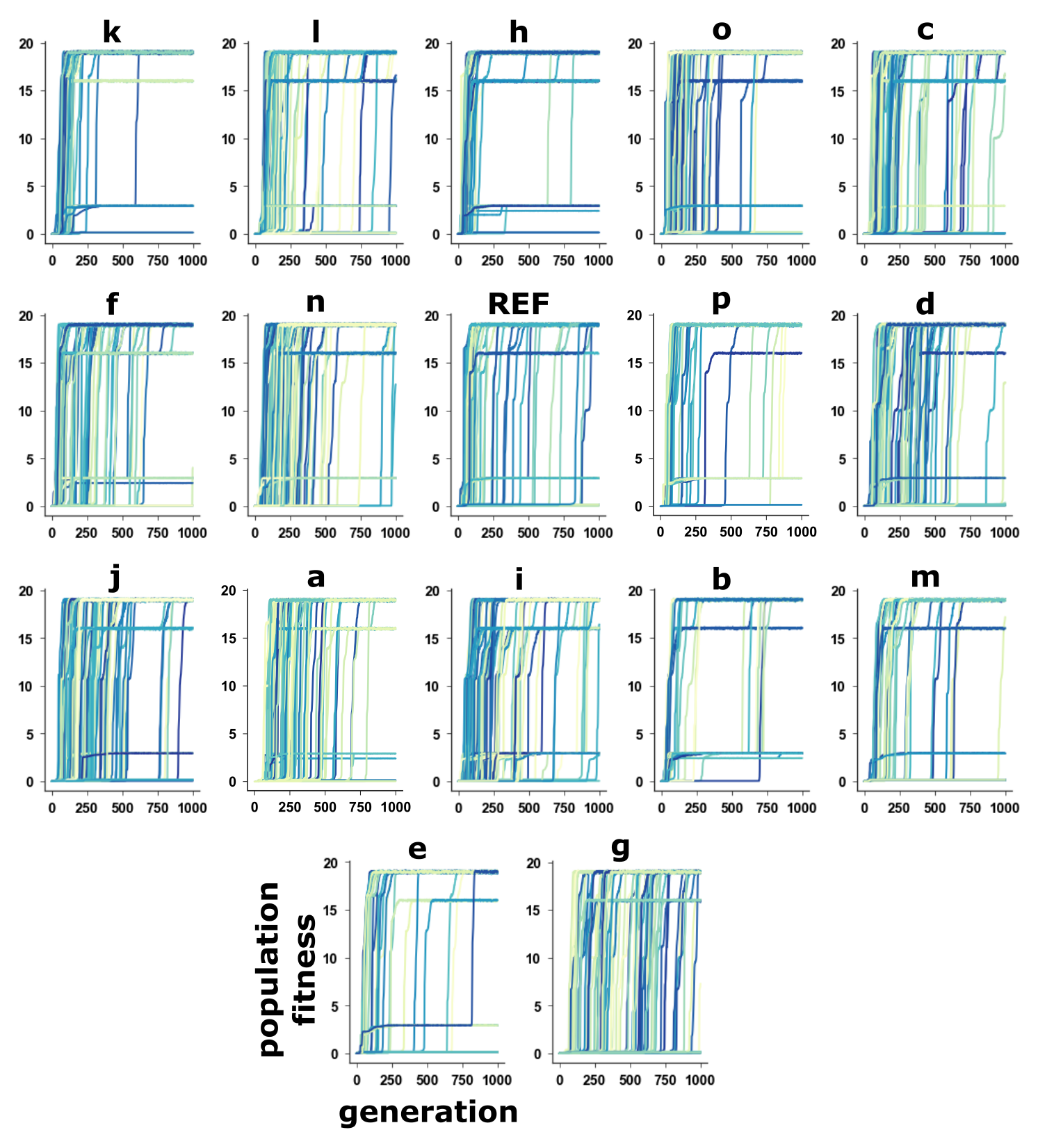

Supplement: S9 Fig — Each trace shows the increase in population fitness over generation time for a single simulation of 1,000 individuals. Each plot shows 100 simulations starting from the same genotype. All starting genotypes has HDV fitness ≥ 1. The letter above each subplot indicates the starting point from the network, as shown in Fig 3A. Letters were assigned alphabetically based on highest to lowest HDV fitness, and genotype a represents the genotype with the highest measured HDV fitness. The graphs are ordered from fastest to slowest initial rates (Fig 4D). Data and Python scripts for evolutionary simulations can be found on GitLab. HDV, Hepatitis Delta Virus. (PNG) [file pbio.3000300.s009.png]

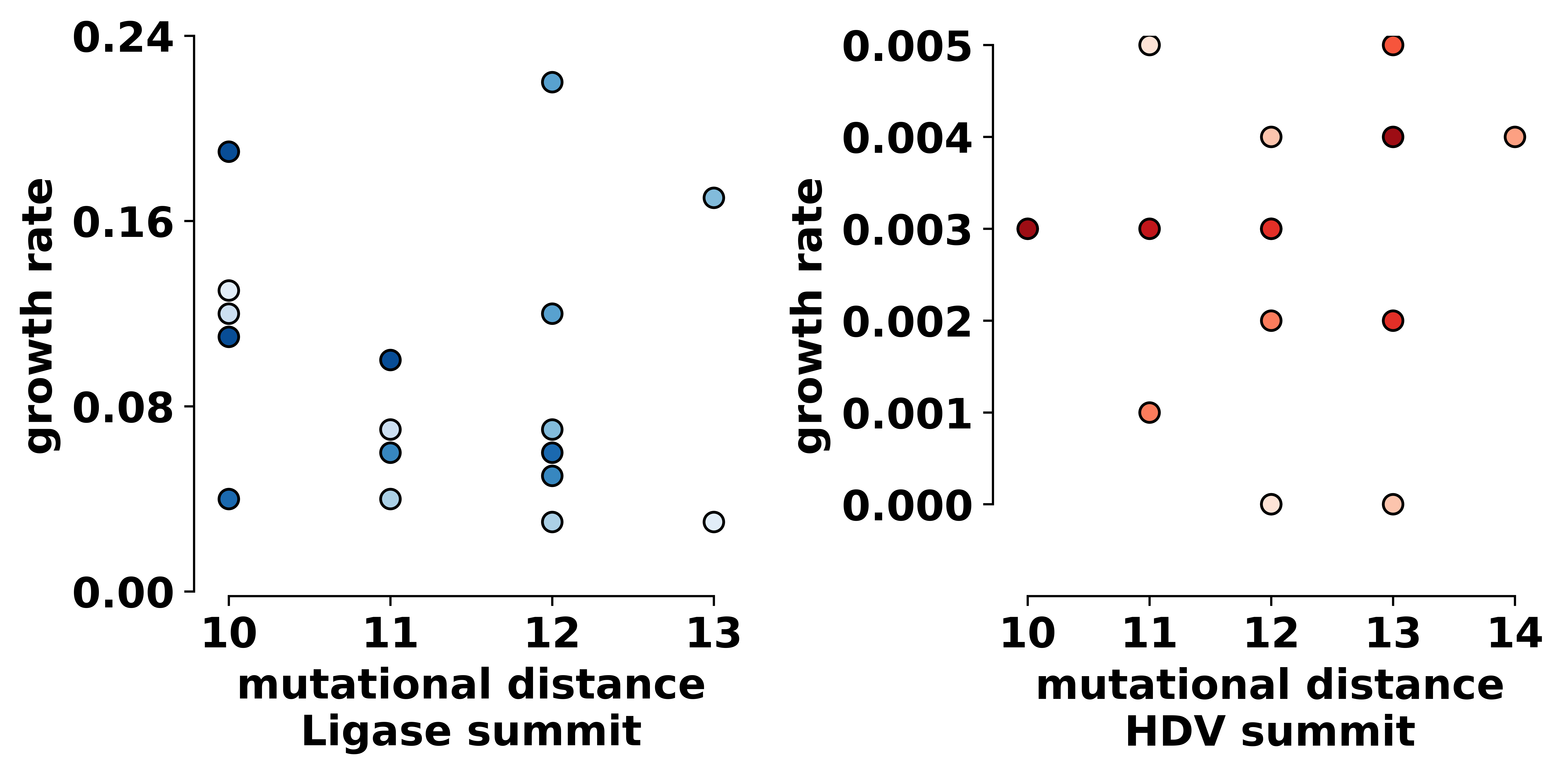

Supplement: S11 Fig — Mutational distance is calculated as the number of mutations between the summit genotype and a given starting genotype for the evolutionary simulations (Fig 4). Data and Python scripts for the relationship can be found on GitLab. (PNG) [file pbio.3000300.s011.png]

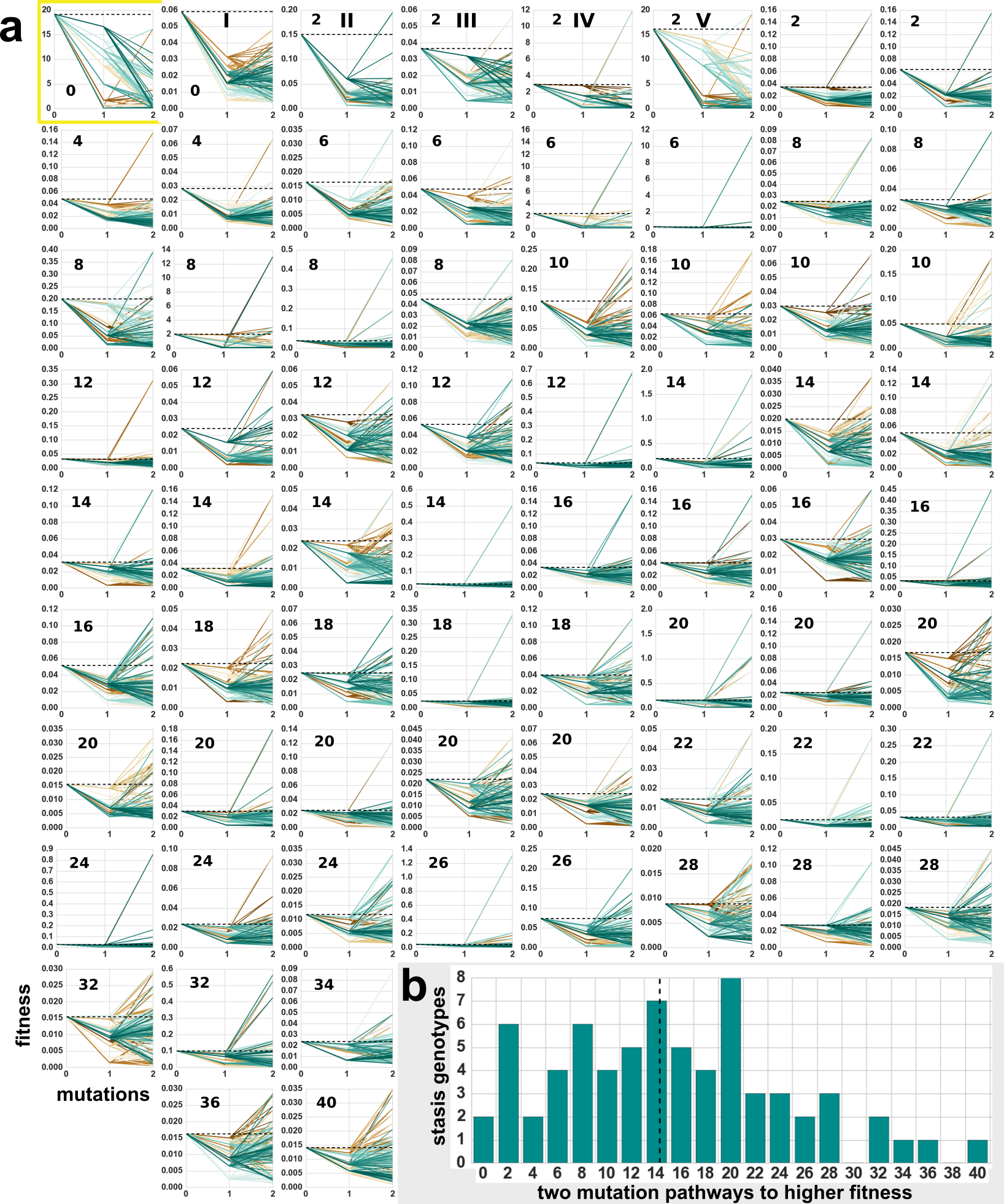

Supplement: S12 Fig — (A) Each line leads from the stasis genotype (mutations = 0) to 1 and 2 mutations away. All 69 stasis genotypes (peaks) in the Ligase fitness landscape are depicted. The number on each graph represents the number of 2-mutation pathways to higher fitness from each stasis genotype. The yellow box indicates the genotype with the highest measured Ligase fitness. Data and Python scripts for calculation and plotting of mutational pathways can be found on GitLab. (B) The distribution of 2-mutation pathways to higher-fitness genotypes from each stasis genotypes in the Ligase landscape. The dotted vertical line indicates the mean of the distribution. (PNG) [file pbio.3000300.s012.png]

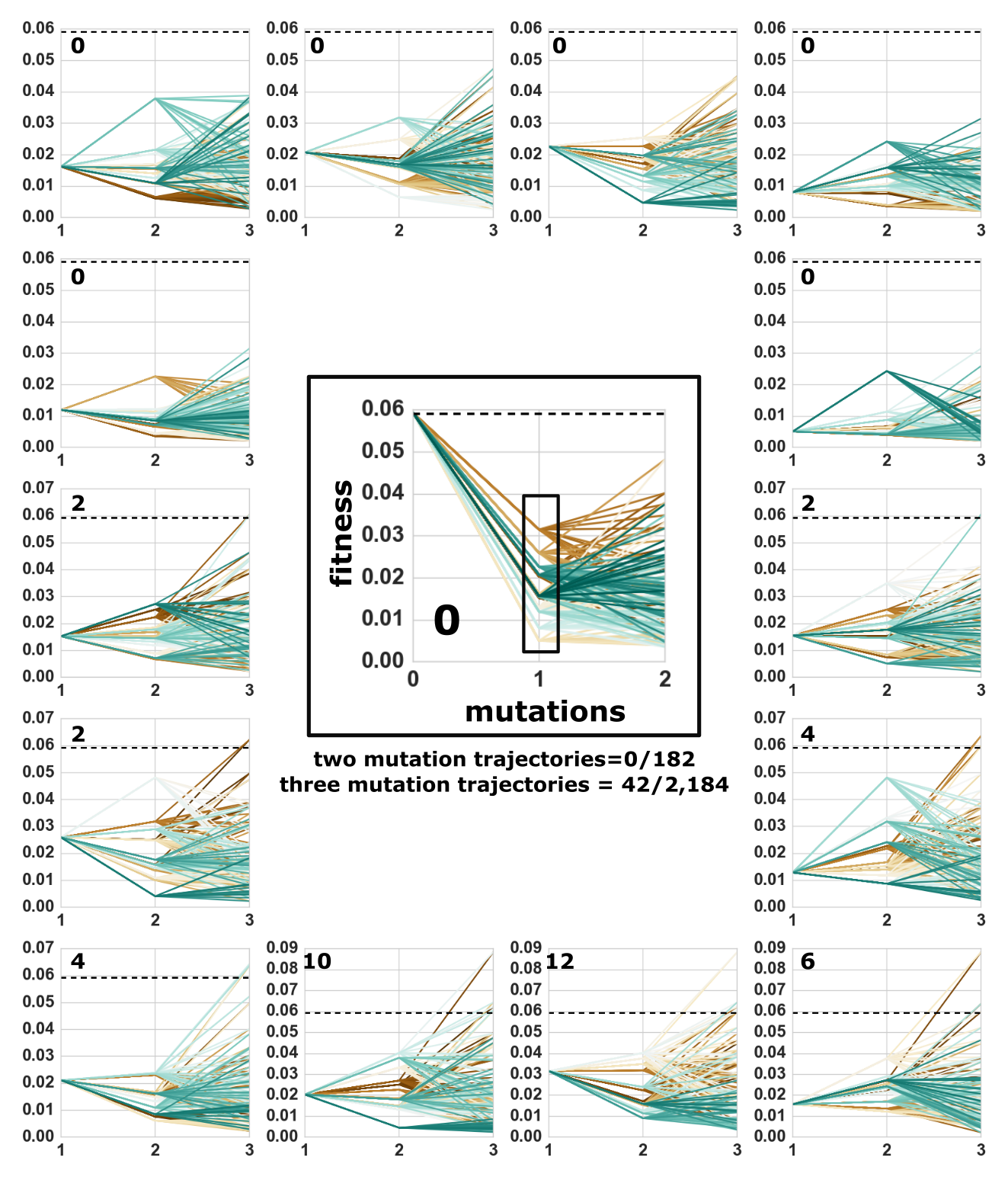

Supplement: S13 Fig — Stasis genotype I from Fig 4F is depicted in the center with each of the 2 mutation trajectories. None of the 182 2-mutation trajectories lead to higher fitness than the stasis genotype (mutation = 0). The pathways 2 mutations from each of the 14 genotypes that are a single mutation away from the stasis genotype are individually depicted. In total, 42 out of a possible 2,184 3-mutation trajectories yield a higher fitness than the initial stasis genotype (dashed line). Data and Python scripts for calculation and plotting of mutational pathways can be found on GitLab. (PNG) [file pbio.3000300.s013.png]

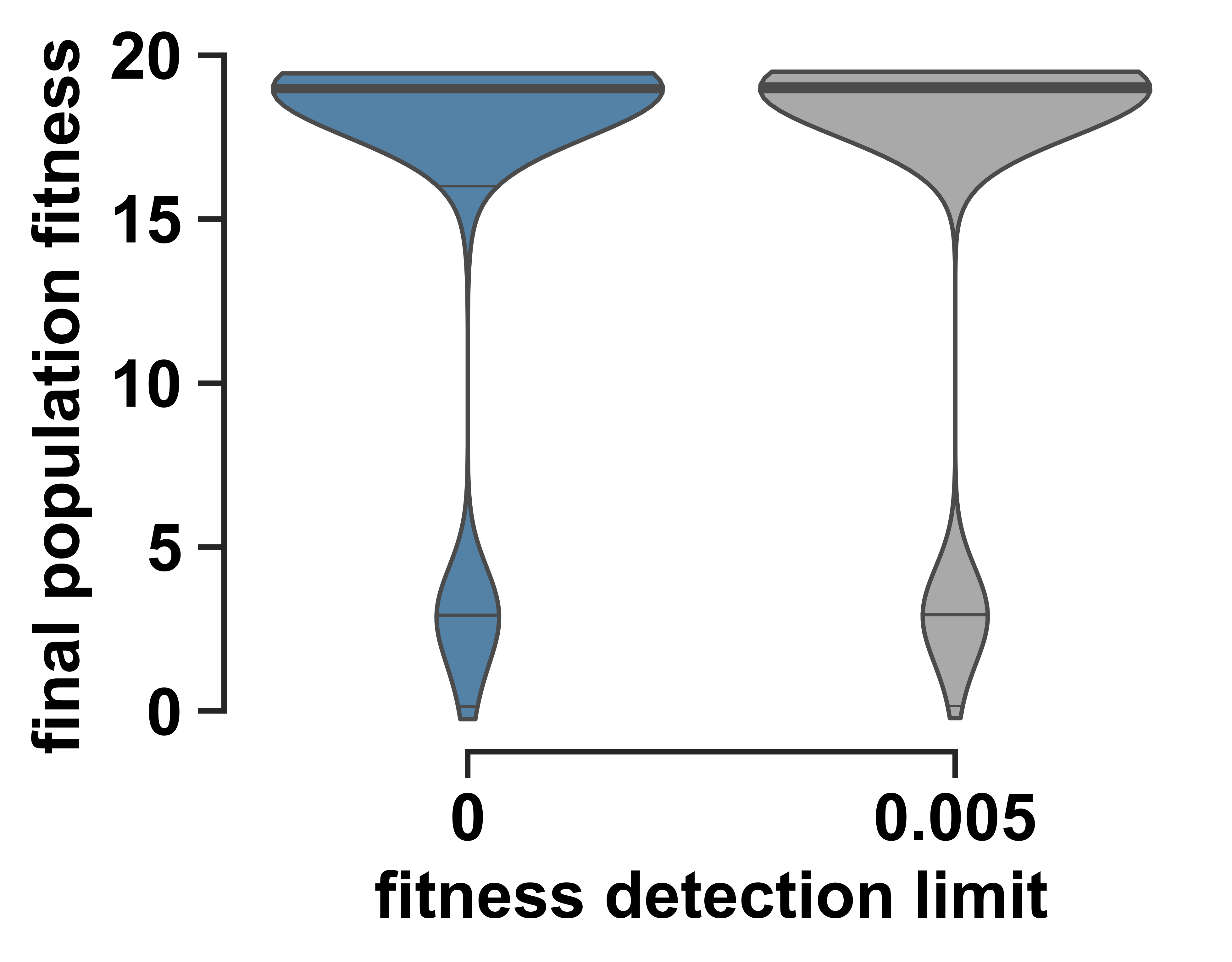

Supplement: S14 Fig — Final fitness of 100 replicate simulations on the original Ligase landscape (red) or a landscape where the lowest-fitness genotypes were converted to fitness = 0 (gray), if fitness was less than 0.005 (7,015 genotypes converted to fitness = 0). Simulations were carried out as in Fig 4 of the main text, with 1,000 individuals and 1,000 generations each. Data and Python scripts for evolutionary simulations can be found on GitLab. (PNG) [file pbio.3000300.s014.png]

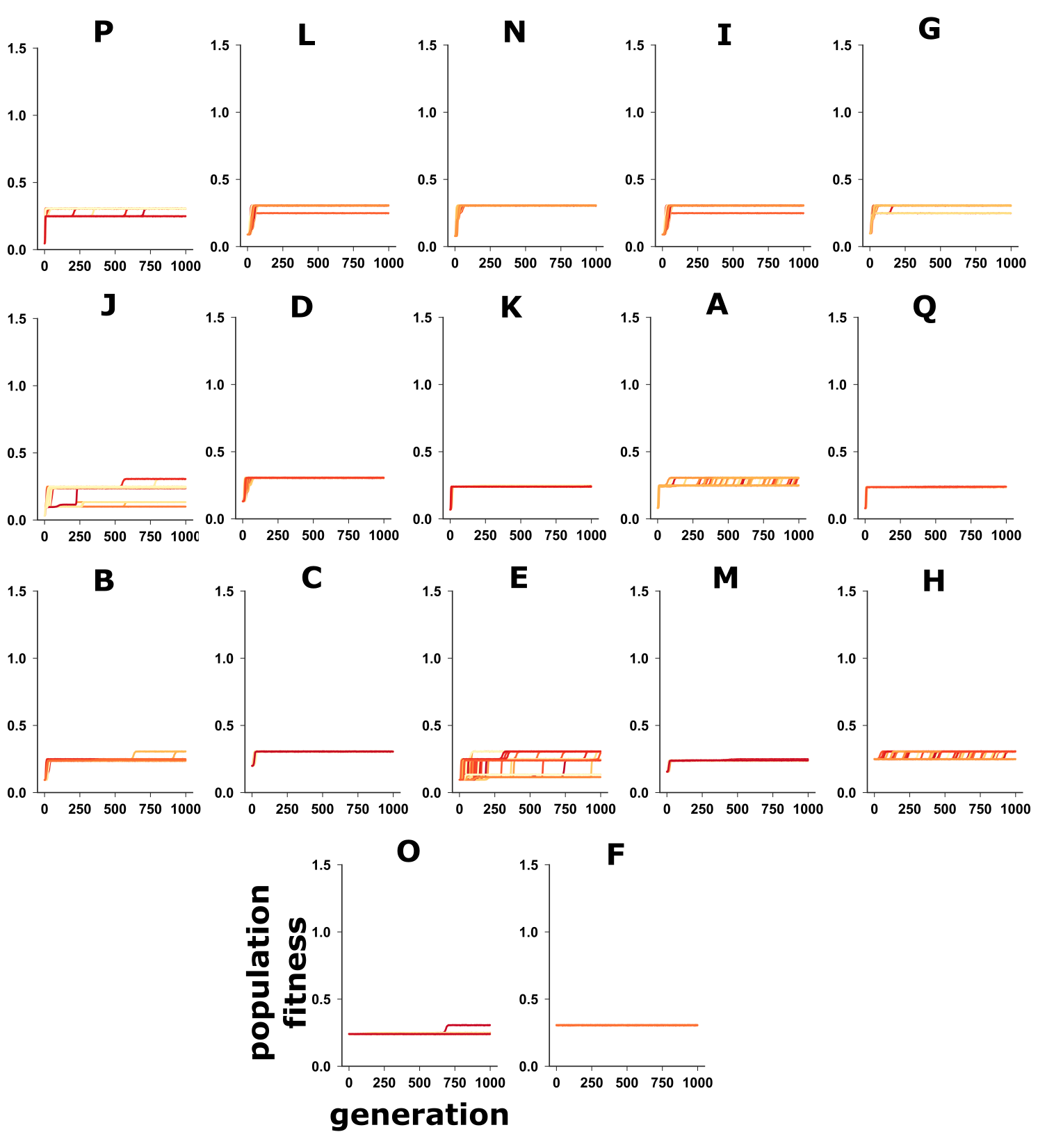

Supplement: S15 Fig — Each trace shows the increase in population fitness over generation time for a single simulation of 1,000 individuals. Each plot shows 100 simulations starting from the same genotype. The letter above each subplot indicates the starting point from the network, as shown in Fig 3A. Letters were assigned alphabetically based on highest to lowest Ligase fitness, and genotype A represents the genotype with the highest measured Ligase fitness. The graphs are ordered from fastest to slowest initial rates (Fig 4E). Data and Python scripts for evolutionary simulations can be found on GitLab. (PNG) [file pbio.3000300.s015.png]

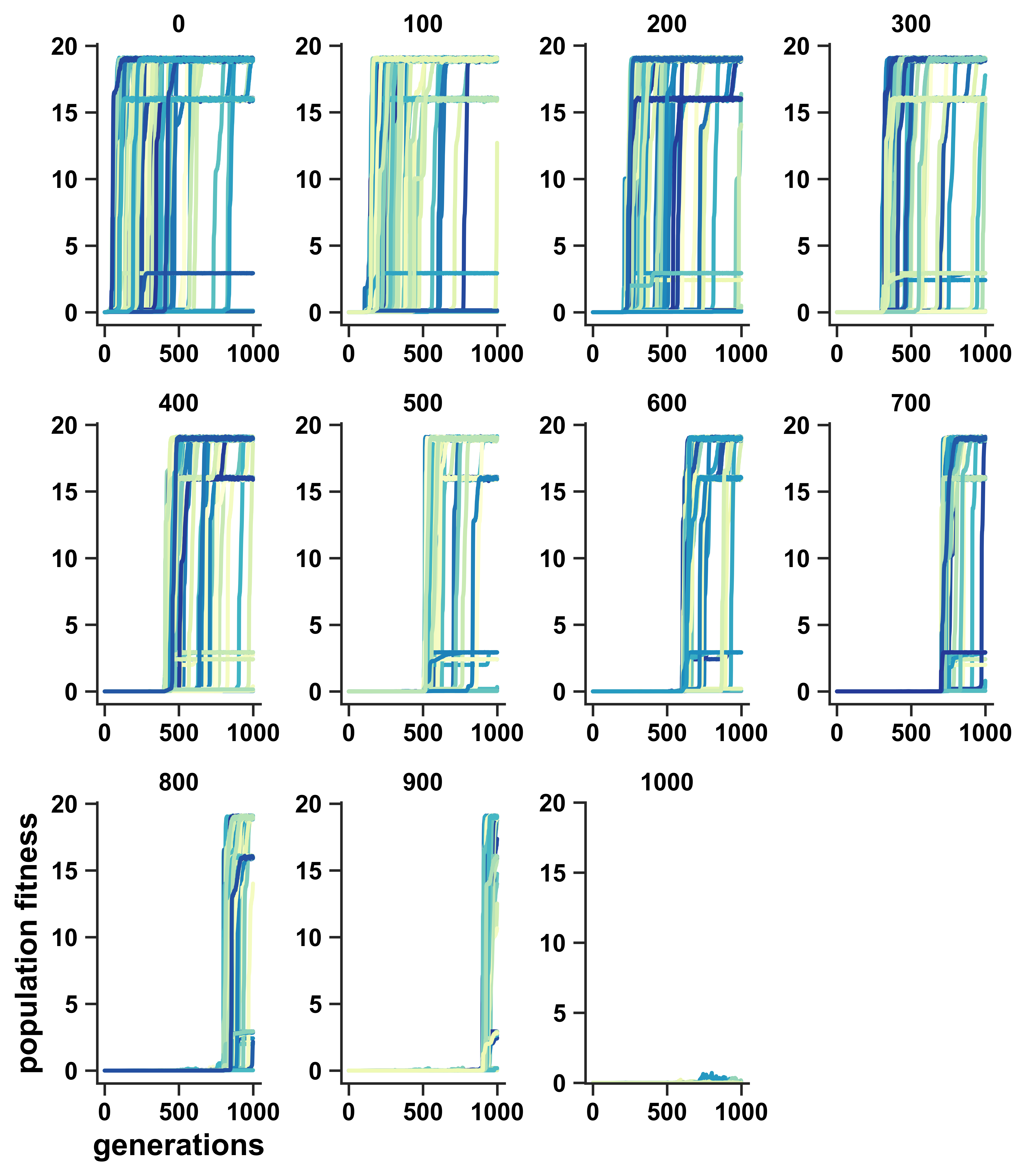

Supplement: S17 Fig — Each trace shows the increase in population fitness over generation time for a single simulation of 1,000 individuals. Each plot shows 100 simulations starting from the summit genotype of the HDV landscape. The number above each subplot indicates the number of generations of neutral evolution before selection was applied as shown in Fig 5. Data and Python scripts for evolutionary simulations can be found on GitLab. HDV, Hepatitis Delta Virus. (PNG) [file pbio.3000300.s017.png]

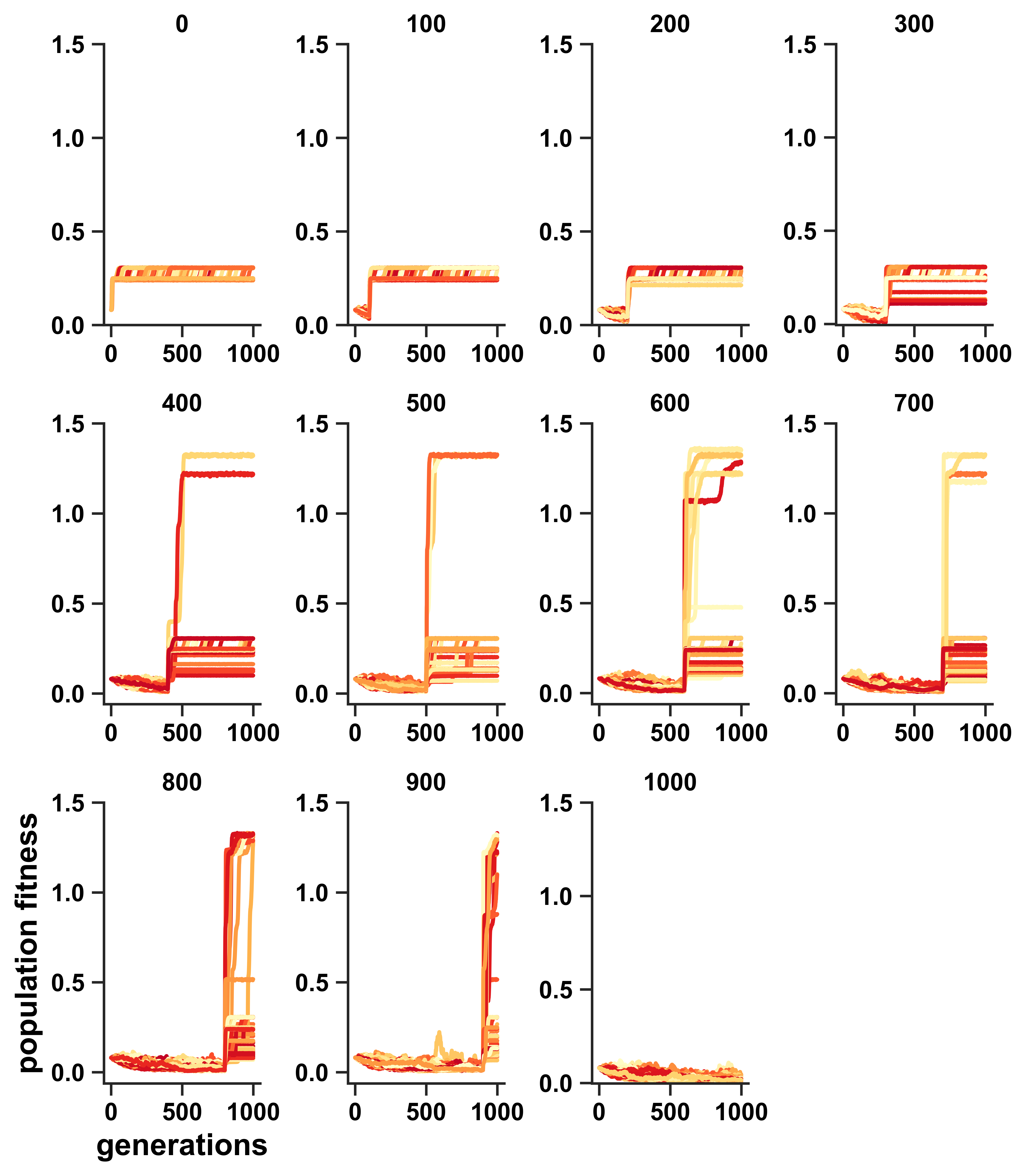

Supplement: S18 Fig — Each trace shows the increase in population fitness over generation time for a single simulation of 1,000 individuals. Each plot shows 100 simulations starting from the summit genotype of the Ligase landscape. The number above each subplot indicates the number of generations of neutral evolution before selection was applied as shown in Fig 5. Data and Python scripts for evolutionary simulations can be found on GitLab. HDV, Hepatitis Delta Virus. (PNG) [file pbio.3000300.s018.png]

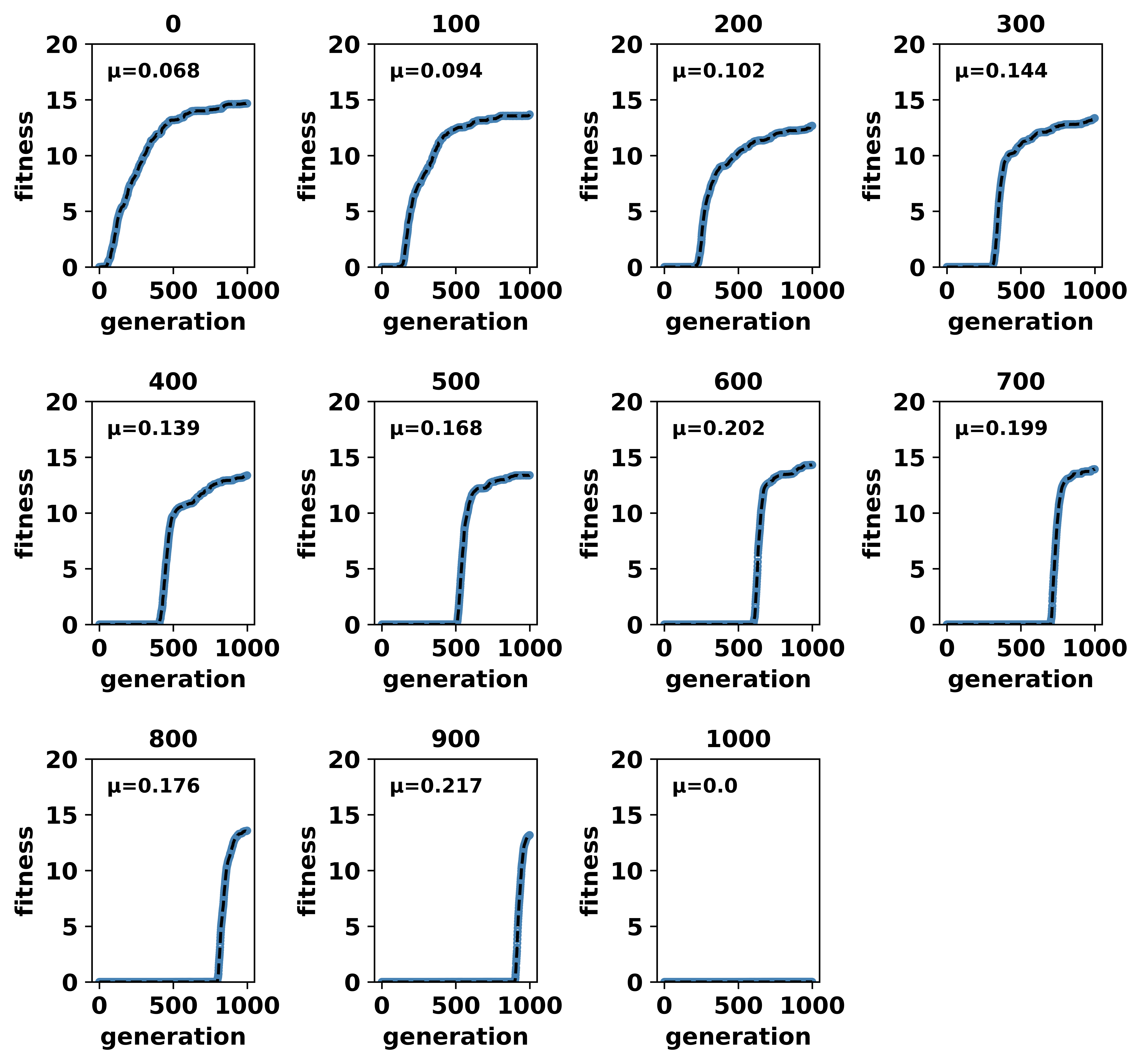

Supplement: S19 Fig — Cubic spline regression was performed for the range of periods of neutral evolution (0–1,000). Solid points are the average of 100 replicates of simulated evolution and correspond to the data from Fig 5A. Dashed black lines are the fitted regression line. The maximum growth rate (μ) derived from the regression is reported on each plot. Data and Python scripts for the regression analysis can be found on GitLab. (PNG) [file pbio.3000300.s019.png]

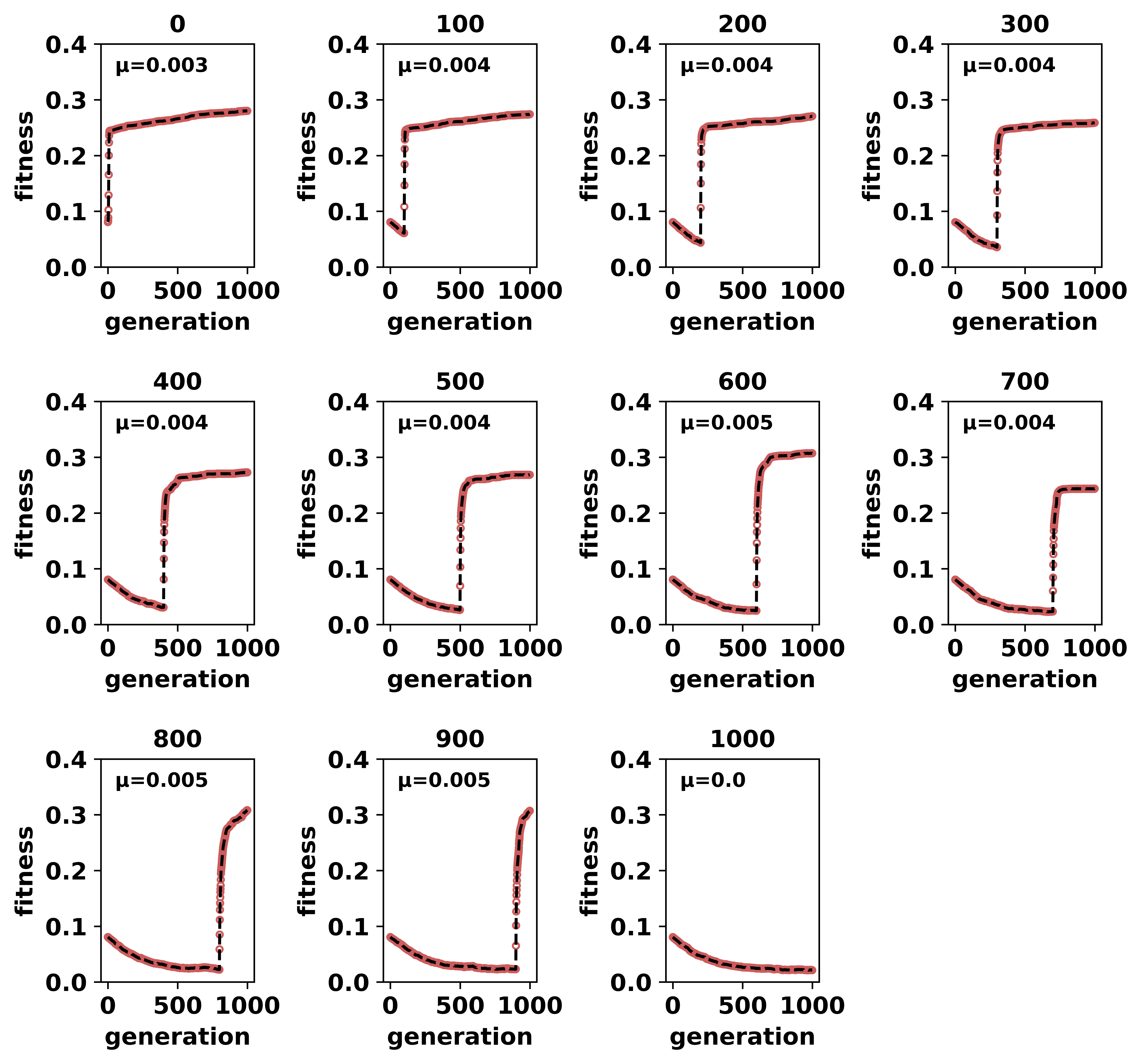

Supplement: S20 Fig — Cubic spline regression was performed for the range of periods of neutral evolution (0–1,000). Solid points are the average of 100 replicates of simulated evolution and correspond to the data from Fig 5B. Dashed black lines are the fitted regression line. The maximum growth rate (μ) derived from the regression is reported on each plot. Data and Python scripts for the regression analysis can be found on GitLab. HDV, Hepatitis Delta Virus. (PNG) [file pbio.3000300.s020.png]

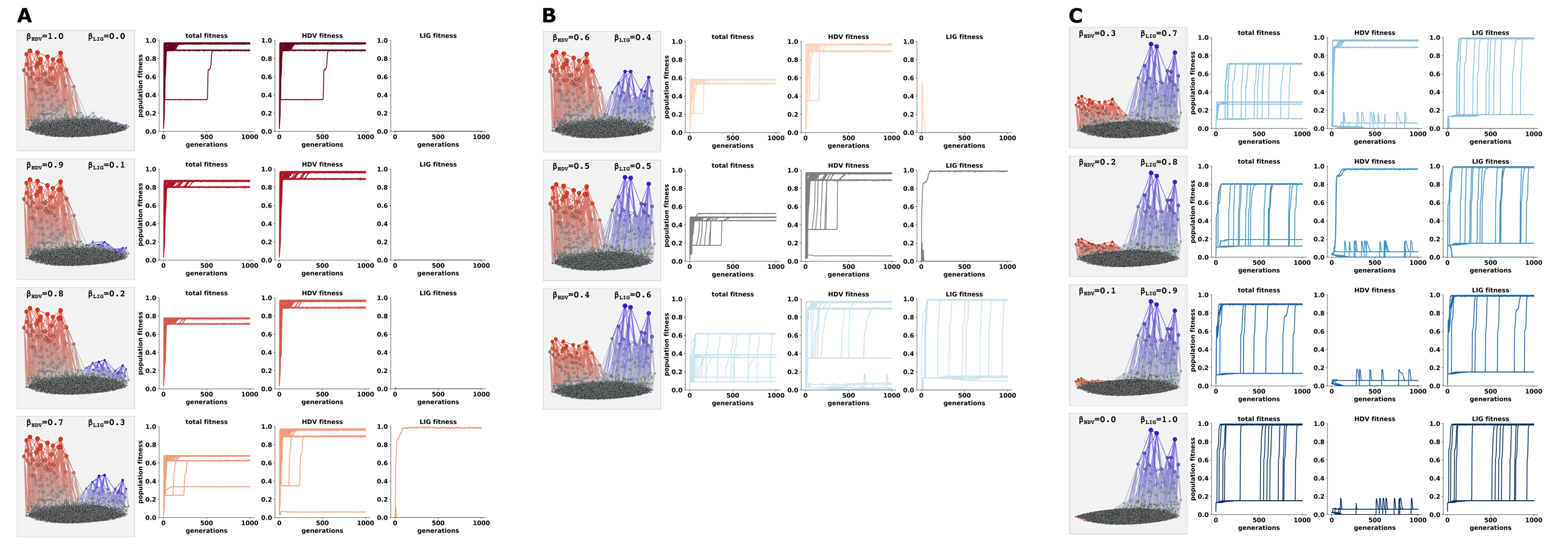

Supplement: S22 Fig — (A–C) Left plot indicates the architecture of the fitness landscape for each combination of weighting parameters (β). Total fitness (calculated as WHDV * βHDV + WLigase * βLigase), HDV fitness, and Ligase fitness are shown for each individual simulation replicate. Color of lines correspond to the weighting parameters discussed in S21 Fig. Data and Python scripts for evolutionary simulations can be found on GitLab. HDV, Hepatitis Delta Virus. (PNG) [file pbio.3000300.s022.png]

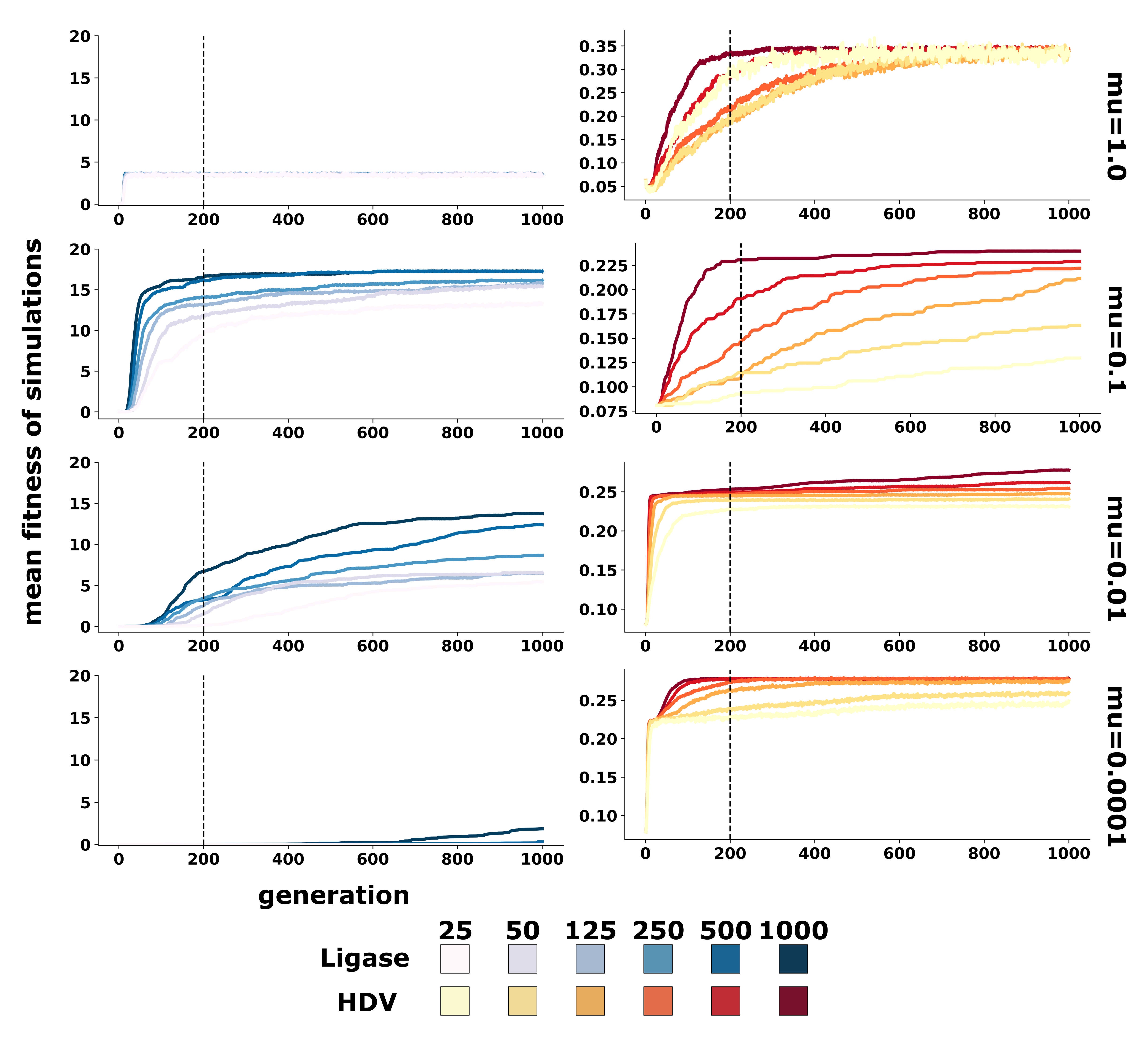

Supplement: S24 Fig — Average rates of evolutionary adaptation of HDV and Ligase activity starting from the summit genotype of the opposing landscape. Trace color indicates the varying population sizes (25–1,000) as indicated in the legend. Each plot indicates a different mutation rate (0.0001–1.0). Each trace shows the mean fitness of 100 simulations as a function of time (generation). The vertical dashed line marks generation 200. Data and Python scripts for evolutionary simulations can be found on GitLab. HDV, Hepatitis Delta Virus. (PNG) [file pbio.3000300.s024.png]

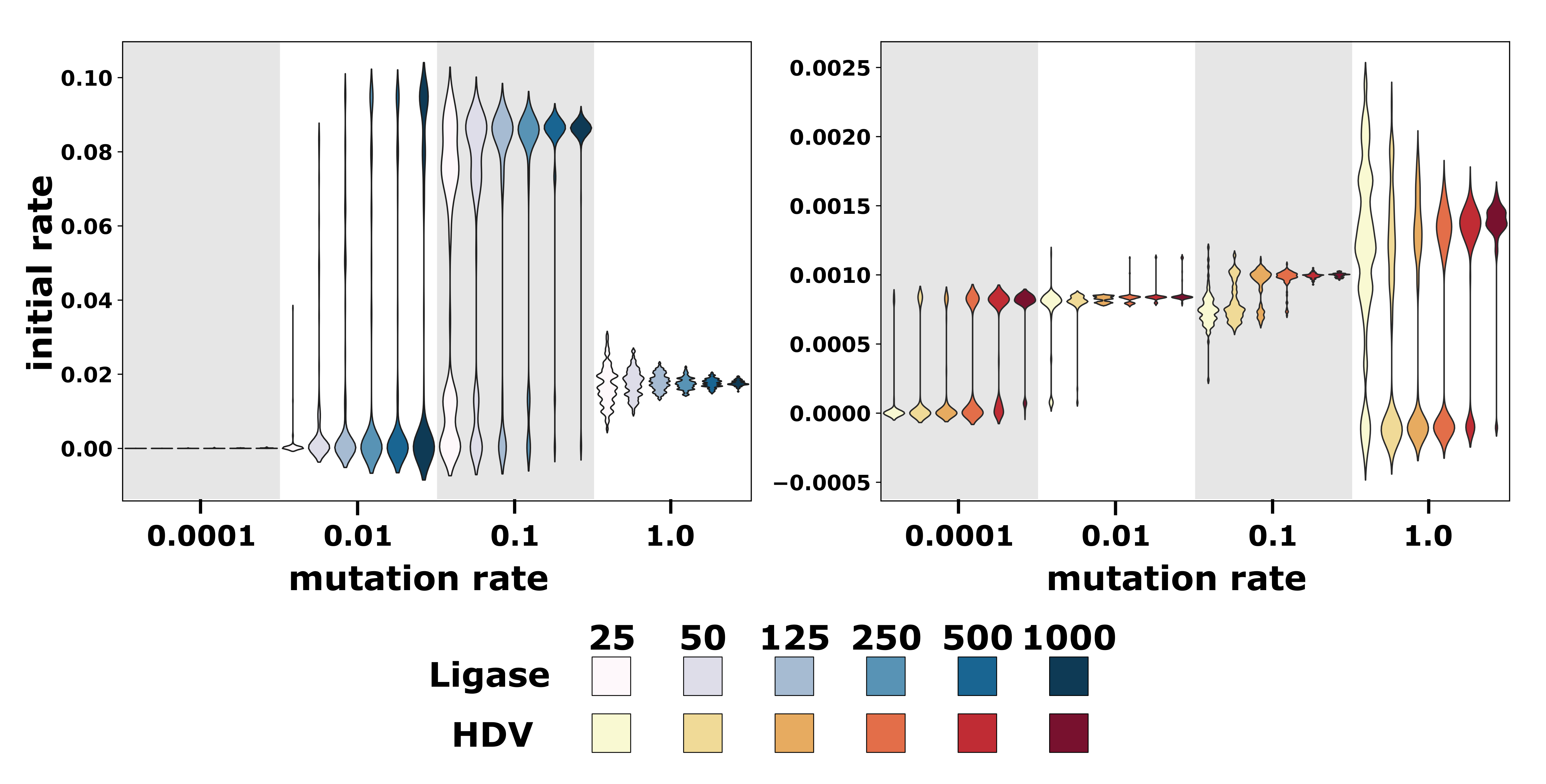

Supplement: S25 Fig — Distributions of initial rates of adaptation on the Ligase and HDV landscape. Initial rate is determined as the rate of population increase for the first 200 generations. Each violin plot represents the distribution of 100 simulations using the same population size and mutation rate. Plot color indicates the varying population sizes (25–1,000) as indicated in the legend. Mutation rate (0.0001–1.0) is indicated on the x-axis. Data and Python scripts for evolutionary simulations can be found on GitLab. HDV, Hepatitis Delta Virus. (PNG) [file pbio.3000300.s025.png]

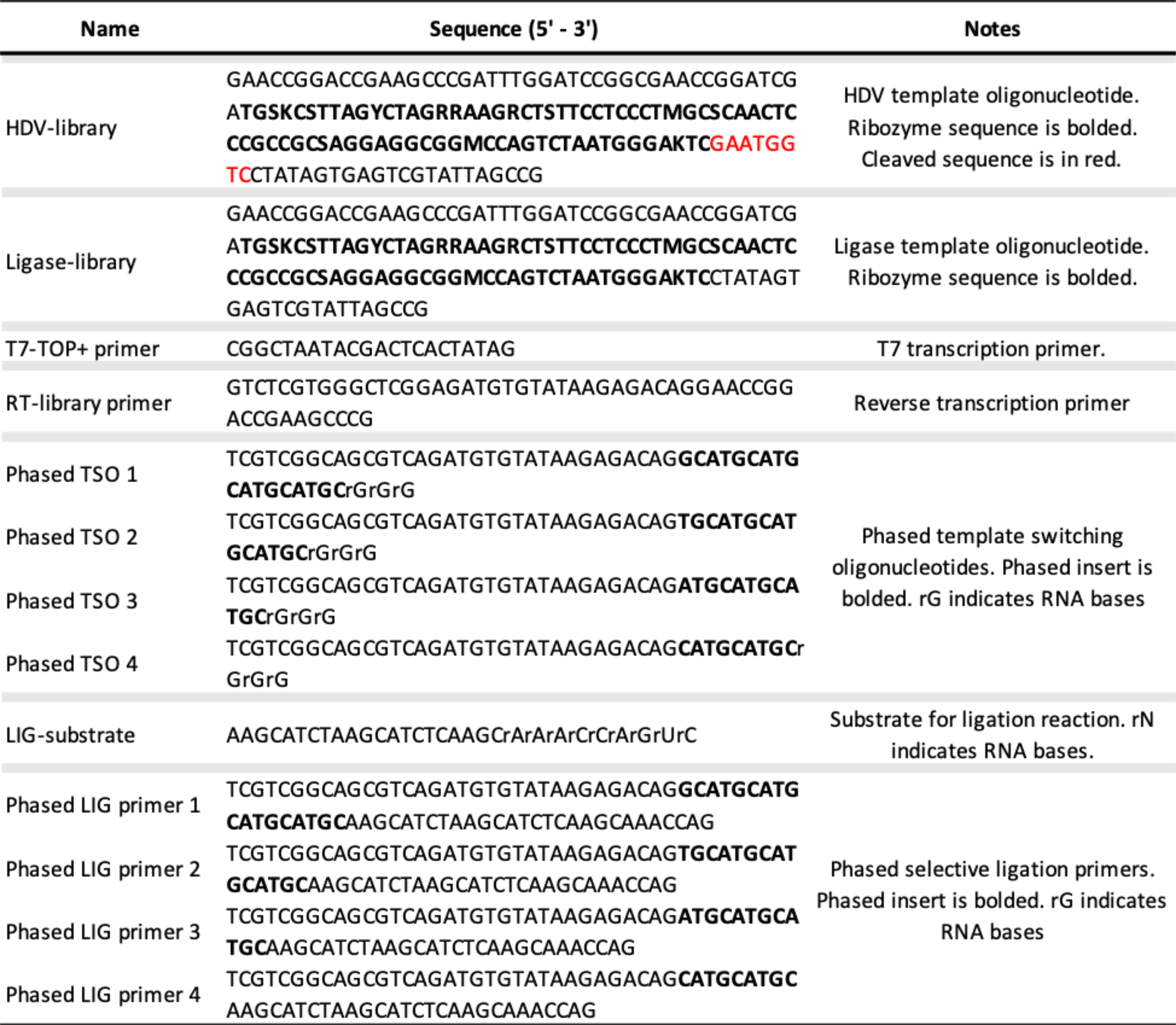

Supplement: S1 Table — (PNG) [file pbio.3000300.s026.png]

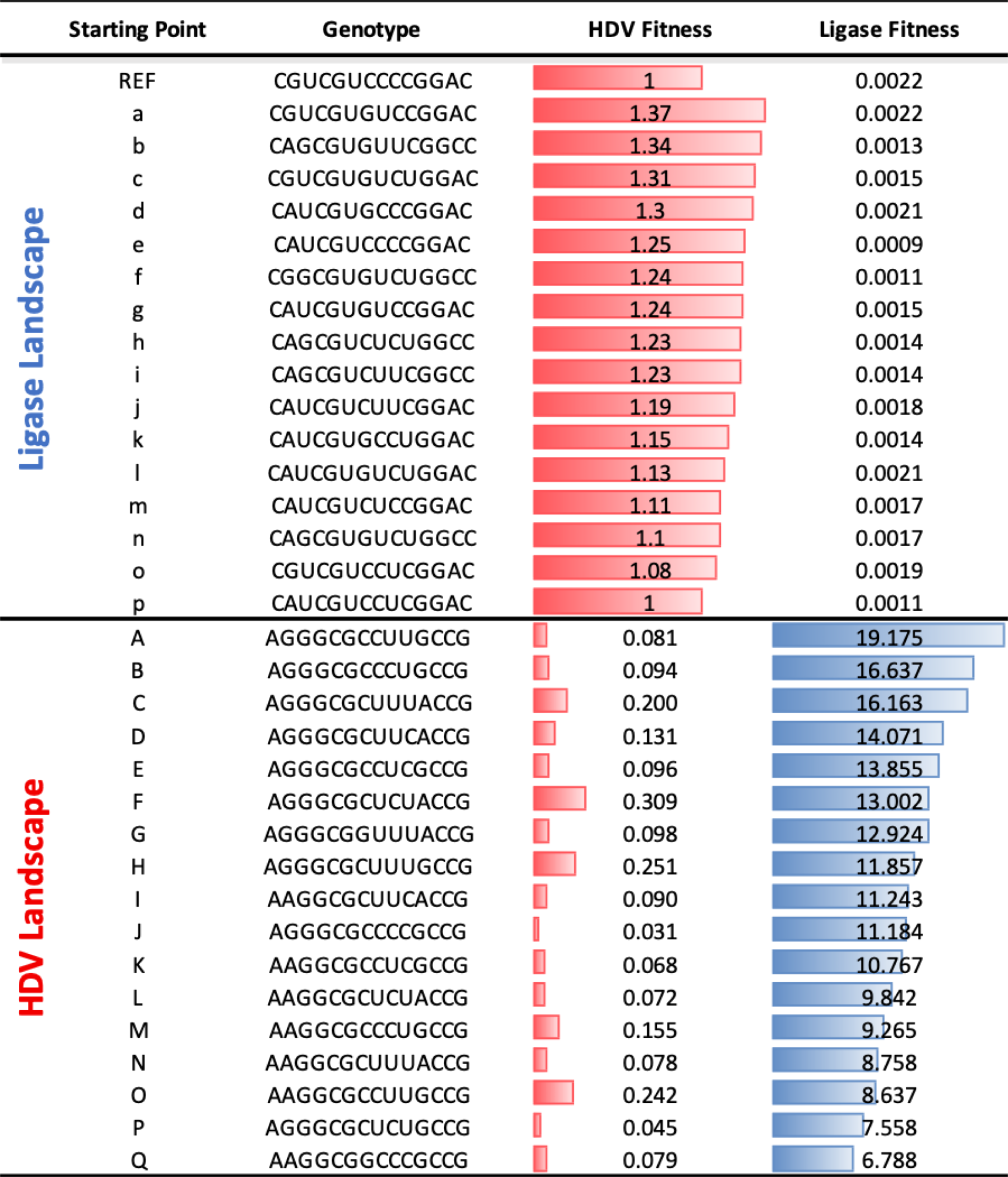

Supplement: S2 Table — Genotypes are represented by the unique combination of nucleotides in the 14 variable positions of the library. Starting point letters correspond to Fig 3A. HDV and Ligase fitness are colored with bar graphs indicating the relative fitness. HDV, Hepatitis Delta Virus. (PNG) [file pbio.3000300.s027.png]
